# Glutathione system enhancement for cardiac protection: pharmacological options against oxidative stress and ferroptosis

**DOI:** 10.1038/s41419-023-05645-y

**Published:** 2023-02-16

**Authors:** Mingyue Tan, Yunfei Yin, Xiao Ma, Jun Zhang, Wanqian Pan, Minghao Tan, Yongjian Zhao, Tianke Yang, Tingbo Jiang, Hongxia Li

**Affiliations:** 1 Department of Cardiology, The First Affiliated Hospital of Soochow University, 188 Shizi Street, Suzhou, 215006 Jiangsu PR China; 2 Department of Nursing, Tianfu College, Southwestern University of Finance and Economics, Chengdu, PR China; 3 Department of Ophthalmology, Eye Institute, Eye & ENT Hospital, Fudan University, Shanghai, PR China; 4 Department of Ophthalmology, The First Affiliated Hospital of USTC, University of Science and Technology of China, Hefei, PR China

**Keywords:** Target identification, Preclinical research

## Abstract

The glutathione (GSH) system is considered to be one of the most powerful endogenous antioxidant systems in the cardiovascular system due to its key contribution to detoxifying xenobiotics and scavenging overreactive oxygen species (ROS). Numerous investigations have suggested that disruption of the GSH system is a critical element in the pathogenesis of myocardial injury. Meanwhile, a newly proposed type of cell death, ferroptosis, has been demonstrated to be closely related to the GSH system, which affects the process and outcome of myocardial injury. Moreover, in facing various pathological challenges, the mammalian heart, which possesses high levels of mitochondria and weak antioxidant capacity, is susceptible to oxidant production and oxidative damage. Therefore, targeted enhancement of the GSH system along with prevention of ferroptosis in the myocardium is a promising therapeutic strategy. In this review, we first systematically describe the physiological functions and anabolism of the GSH system, as well as its effects on cardiac injury. Then, we discuss the relationship between the GSH system and ferroptosis in myocardial injury. Moreover, a comprehensive summary of the activation strategies of the GSH system is presented, where we mainly identify several promising herbal monomers, which may provide valuable guidelines for the exploration of new therapeutic approaches.

## Facts

- GSH system is one of the important endogenous antioxidant systems, which can maintain cellular redox balance and prevent oxidative damage and cell death.
- Ferroptosis is a new form of programmed cell death that plays an essential role in the development of cardiovascular disease.
- The GSH system, as a lipid peroxide scavenger, prevents the indefinite expansion of lipid peroxidation, which is a central step in hindering ferroptosis.
- Activating the GSH system alleviates the progression of the myocardial injury by blocking the ferroptosis pathway and oxidative stress, which provides a promising therapeutic strategy for cardiac diseases.

## Open questions

- How GSH anabolism is altered in the myocardium under pathological and physiological conditions.
- What is the relationship between the GSH system and ferroptosis in various types of myocardial injury.
- Are there therapeutic agents that act to activate the GSH system to reduce myocardial injury.

## Introduction

Cardiovascular diseases (CVDs) are the leading cause of death worldwide and rising health care costs [1]. Irreversible heart injury is the main culprit for the poor prognosis of CVDs. Therefore, understanding how cardiac tissue is impaired is critical to global health. Oxidative stress plays an essential and complex role throughout its occurrence and progression [2–6], to which reactive oxygen species (ROS) are the primary contributors. They consist of a group of highly active molecules, including free radical superoxide (O_2_^• −^), hydroxyl radical (HO^•^), hydrogen peroxide (H_2_O_2_), proximities (ONOO-) and so on [4]. Under physiological conditions, ROS are generated in the heart mainly from the mitochondrial electron transport chain, nitric oxide synthases (NOS), NADPH oxidases (NOX), and xanthine oxidase (XO) in small quantities [7, 8]. Furthermore, the production and degradation of ROS are in dynamic equilibrium. Under pathological conditions, the mitochondrial electron transport chain causes the formation of large amounts of ROS. Mitochondrial ROS overproduction has been indicated to donate to cardiomyocyte injury and greater myocardial injury after acute myocardial infarction (AMI) [9, 10]. In addition, the increased expression and activity of NOX and xanthine oxidase and NOS becoming uncoupled and structurally unstable also lead to increased ROS production [8]. Excess ROS can damage all major cellular constituents (DNA, proteins, and lipids) and even lead to cell death, apoptosis, hypertrophy, fibrosis, and contractile dysfunction in the myocardium [8, 11].

For the body to resist these damages, it possesses a range of endogenous antioxidants, primarily enzymatic antioxidants (superoxide dismutase (SOD), glutathione peroxidase (GPX), glutathione S-transferase (GST), glutathione reductase (GR), thioredoxin reductase (TrxR), catalase (CAT), etc.), and nonenzymatic antioxidants (glutathione (GSH), bilirubin, ubiquinone, etc.) [11]. Among them, the glutathione system made up of GSH, enzymes involved in GSH anabolism, and GSH-dependent antioxidant enzymes play a critical role in cardioprotection from oxidative stress and redox homeostasis [12–14]. Due to cardiomyocytes requiring massive energy demands, they are rich in mitochondria standing out from the rest of the cell type [15, 16], which makes them very sensitive to oxidative stress damage. At the same time, the activity of antioxidant enzymes (SOD, GPX, GR) in cardiomyocytes is weaker than that in other organs [17], further aggravating the susceptibility to this injury. Therefore, targeted activation of antioxidant systems, especially the GSH system, is an essential treatment strategy to protect cardiomyocytes against oxidative damage.

Notably, redox signaling events are significant regulators of cell death pathways, among which GSH depletion has been demonstrated to be a vital early hallmark in the progression of different cell death mechanisms [18]. Distinct from apoptosis, necrosis and autophagy, Dixon and coauthors proposed for the first time a new form of oxidation-dependent and iron-dependent cell death known as ferroptosis [19]. It is characterized by an extremely severe accumulation of lipid peroxidation [20]. Numerous studies have shown that GSH anabolism and GPX4 are inextricably linked to ferroptosis. Toxic lipid peroxides can be converted into nontoxic lipid alcohols (L-OH) in the presence of GPX4 and GSH to inhibit ferroptosis [21]. However, some molecules or drugs, such as erastin, sulfasalazine, sorafenib, and RSL3, are able to block glutathione synthesis or devitalize GPX4. All of these processes tend to initiate ferroptosis, leading to cell damage [22].

**Fig. 1:**
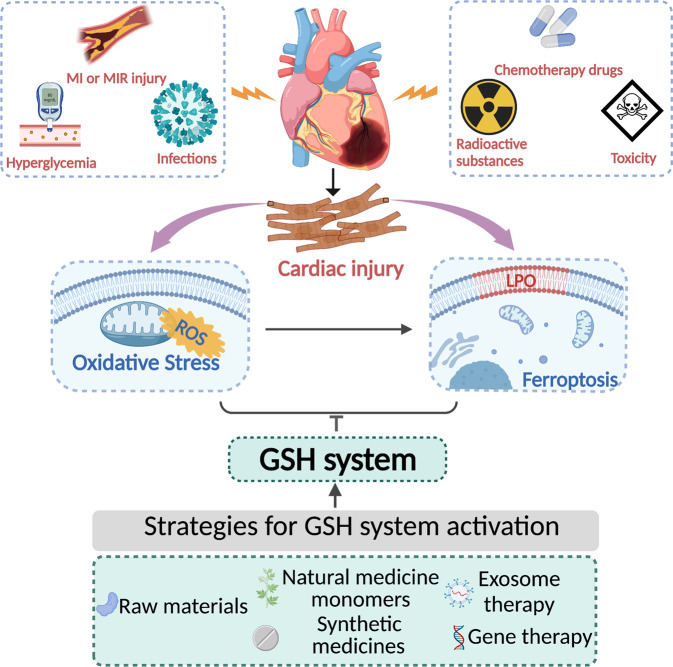
Strategies to augment the cardiac GSH system during myocardial injury. Raw materials of GSH or GPX biosynthesis, various small molecule activators related to the GSH system and some novel therapeutic approaches, such as exosomes and gene therapy, enhance the GSH system and thus improve myocardial injury caused by various pathological factors by inhibiting oxidative stress and ferroptosis.

In summary, oxidative stress drives the occurrence and progression of CVDs, and the GSH system is positively involved in antioxidant activity, which is beneficial to their treatment and prognosis. Current studies have revealed a strong link between ferroptosis and the GSH system, which has become a hotspot in the field of CVDs. As a result, herein, we review the synthetic and metabolic processes of GSH, systematically describing the functions of the GSH system in cardiac injury. Moreover, we emphasize GSH system-related ferroptosis in cardiac injury events and comprehensively summarize strategies for GSH system activation. Understanding the roles and interventions of the GSH system, as well as its connection with ferroptosis, may favor the advancement of more effective therapies to prevent myocardial injury (summarized in Fig. 1).

## Overview of the GSH system

### The anabolic process and redox status of GSH in the myocardium under physiological conditions

#### Synthesis of GSH

The synthesis of glutathione is found in almost all mammalian cells [23]. This process, using glutamate (Glu), cysteine (Cys), and glycine (Gly) as raw materials, depends on glutamate cysteine ligase (GCL) and glutathione synthetase (GS) catalysis, present only in the cytoplasmic matrix [23]. Its rate-limiting steps are the availability of cysteine and the activity of GCL [24]. The three amino acids mentioned above come from different pathways. Cysteine is mainly derived from the trans-sulfuration pathway of methionine and the reduction of cysteine [21]. In the former, cysteine comes from the conversion of methionine via a series of enzymatic steps, which is unique to liver cells [25]. In the latter, cysteine originates from the reduction of cystine transported into the cell by cystine/glutamate antiporter (system Xc − , xCT) [26]. The system Xc−, a dimer consisting of SLC7A11 and SLC3A2, exchanges glutamate (Glu) out of the cell and cystine into the cell at a ratio of 1:1 [27, 28]. Among them, SLC7A11 has a key role in limiting the rate of cystine transport [27]. Intracellular Glu is produced from glutamine (Gln) catalyzed by glutaminase (GLS, with two different isomers GLS1 and GLS2) [29, 30]. Gln and Gly are transported into the cell via the corresponding amino acid transporters. In addition, glycine and glutamate deficiency also affects GSH synthesis to some extent [23].

**Fig. 2:**
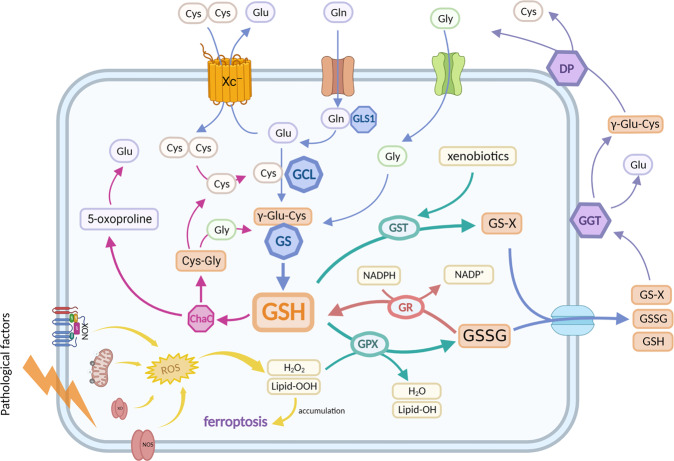
Schematic diagram of intracellular GSH anabolism and function. The main synthetic pathways of GSH (blue arrows); GSSG is reduced to GSH under the action of GR and NADPH (red arrow); GSH reduces H_2_O_2_ (or lipid-OOH) to H_2_O (or lipid-OH) in the catalysis of GPX, and GSH conjugates with xenobiotics compounds to form GS-X in the catalysis of GST (green arrow); The decomposition process of GSH, GSSG and GS-X after being squeezed out of cells (purple arrows); The decomposition process of intracellular GSH (rosy arrows); ROS are generated from the mitochondrial electron transport chain, nitric oxide synthases (NOS), NADPH oxidases (NOX), and xanthine oxidase (XO) (yellow arrow).

The synthesis of glutathione involves two enzymatic steps, both requiring ATP hydrolysis for energy supply. First, glutamate-cysteine ligase (GCL) links glutamate and cysteine to form γ-glutamyl cysteine (rate-limiting steps). Glutathione synthetase (GS) then catalyzes the combination of glycine residues and gamma-glutamyl-cysteine to form GSH [31]. GCL is composed of a catalytic (GCLC, Mr ~73 kDa) and a modifier (GCLM, Mr ~31 kDa) subunit [32] (summarized in Fig. 2).

#### Metabolism of GSH

Glutathione, the most plentiful thiol-containing substance of low molecular weight in cells, is a crucial antioxidant and antidote in all mammalian tissues. Under physiological conditions, GSH quenches oxidizing substances (such as reactive hydroxyl free radicals, peroxynitrite, and H_2_O_2_) directly or reduces hydrogen peroxide (or lipid peroxide (lipid-OOH)) to water (or the corresponding lipid alcohol (lipid-OH)) under the catalysis of GPX (summarized in Fig. 2). Meanwhile, reduced GSH is oxidized into glutathione disulfide (GSSG) [8, 32]. GSH also detoxifies electrophilic xenobiotics compounds (chemical carcinogens, environmental pollutants, drugs, etc.) and their metabolites to GSH adduct (GS-X) in the direct catalysis of glutathione-S-transferase (GST, primary approach) [32]. The concentration of GSH is at high levels, and GSSG and ROS are at depressed levels to maintain redox homeostasis in cardiomyocytes. Thus, the ratio of GSH to GSSG is largely considered a marker of oxidative stress [8, 32].

To maintain high levels of GSH in cardiomyocytes, there are generally two mechanisms. One is that GSSG and GS-X produced in cardiomyocytes are pumped out by multidrug resistance protein 1 (MRP1), preventing GSSG or GS-X accumulation in cardiomyocytes [33, 34], and subsequently enter the γ-glutamyl cycle. This cycle primarily involves γ-glutamyl peptidase (GGT), the only enzyme that catabolizes GSH, GSSG, and GS-X, as well as dipeptidyl peptidase (DP). GGT transfers the γ-glutamyl residue of glutathione to amino acid acceptors (the best acceptor being cystine), releasing γ-glutamyl peptides and cysteinyl glycine. Subsequently, DP breaks cysteinyl glycine into cysteine and glycine, which are reabsorbed into the cell to engage in a novel GSH synthesis pathway (summarized in Fig. 2) [31, 35]. The other is that some GSSG can be reduced to GSH in the presence of GR at the cost of NADPH [8] (summarized in Fig. 2). NADPH is mainly derived from the pentose phosphate pathway, which uses glucose-6-phosphate dehydrogenase (G6PD) as the key enzyme [36]. The ChaC family (ChaC1 and ChaC2, cytoplasmic glutathione-specific γ-glutamyl cyclotransferases) has recently been shown to take part in GSH metabolism. ChaC1 and ChaC2 use the α-amine of L-glutamyl residues to cleave the amide bond by transamination, releasing it as a cyclic 5-oxo-l-proline and cysteine glycine dipeptide. These products are cleaved into glutamine, cysteine, and glycine under the action of 5-oxoalaninase and Cys-Gly peptidase, respectively, and then added to new GSH synthesis [32, 37] (summarized in Fig. 2). However, the role of the ChaC family in cardiomyocytes has been less reported since then.

### The anabolic variety and redox status of GSH in the myocardium under pathological conditions

When the heart is under stress from pathological factors, GSH redox homeostasis is disrupted, which is manifested by a significant decrease in GSH content and accumulation of GSSG in the myocardium. Several clinical studies have found that GSH consumption exists in the blood of patients with myocardial infarction and the left ventricle of patients with heart failure [8, 38, 39]. Moreover, a reversed GSH redox state and activity-suppressed GPX have been observed in the myocardium of various myocardial injury animal models [40–43]. The reasons for these changes, apart from excess ROS leading to increased GSH depletion in cardiomyocytes, are disturbances in the anabolic process of GSH, causing a further decrease in intracellular GSH content. Many preclinical studies have demonstrated that the expression of key enzymes for GSH synthesis (GCL, SLC7A11, GS) and GSSG reduction-related enzymes (G6PD, NADPH, and GR) is downregulated in myocardium exposed to pathological factors [40, 43–50]. However, enhanced expression of GGT and MRP1, which are associated with the γ-glutamyl cycle, has been observed in damaged myocardium [33, 34, 51, 52]. Moreover, some studies have shown that the ChaC1 protein is significantly upregulated in various adverse conditions, such as diabetes, viral infection and atherosclerosis, providing cells with the amino acid nutrients they need under stressful situations but reducing GSH levels. However, the ChaC family is less studied in the myocardium [37]. Hiroki Kitakata et al. reported that in MITOL knockdown-mediated ferroptosis in cardiomyocytes, knockdown of ChaC1 reversed cell injury and increased GSH [53]. ChaC2 is a constitutively expressed protein whose expression is not affected by the external environment for the basal turnover of GSH [37].

### GSH in mitochondria

As the most metabolically demanding organ in the body, the heart requires significant amounts of energy adenosine triphosphate (ATP) to maintain constant contractile and diastolic function. Therefore, it is not surprising that cardiomyocytes have a high volume of mitochondria (30-40% of the entire cell) [54]. In addition, mitochondria are also the primary producers of intracellular reactive oxygen species (ROS), most of which originate from the mitochondrial respiratory chain [55]. In accordance with this, the steady-state concentration of O_2_^• −^ in the mitochondrial matrix is estimated to be five to ten times higher than that in the cytosol [56]. Despite the exposure of mitochondria to the production of oxidants, the presence of an effective antioxidant system, of which mitochondrial GSH (mGSH) is a key component, prevents or repairs oxidative damage that occurs during normal aerobic metabolism [57]. The importance of mGSH is mainly based on its detoxification of hydrogen peroxide, mitochondrial lipid membrane peroxidation, or xenobiotics under the catalysis of enzymes such as GPX or GST [58]. Thus, as far as mitochondrial oxidative stress homeostasis is concerned, mGSH is a vital factor maintaining mitochondrial function, controlling cell survival/death, and its depletion causes cell damage and facilitates cell death [57, 59]. A recent report suggests that mGSH and mitochondrial redox status play an essential role in cardiomyocyte ferroptosis [60].

As mentioned above, GSH is synthesized only in the cytoplasmic matrix, but it is also found in intracellular organelles, including the mitochondria, nucleus, and endoplasmic reticulum. Mitochondria contain 10–15% of total GSH, and the concentration of GSH in mitochondria is similar to that of the cytoplasmic matrix [57, 58]. However, GSH has an overall negative charge at physiological pH, and mitochondria also exhibit an enormous negative membrane potential [57, 58]. Therefore, mGSH arises from cytoplasmic matrix GSH via a specific transport mechanism. Some investigators have identified potential candidates for transporting GSH into the kidney and liver mitochondria, including the 2-oxoglutarate carrier (OGC; SLC25A11) and the dicarboxylate carrier (DIC; SLC25A10) [57]. In 2021, Zhang et al. found that these two vectors were highly expressed in myocardial mitochondria and showed that inhibition of DIC and OGC increased mitochondrial ROS and GSH depletion, exacerbating ferroptosis in the myocardium [60]. Recently, it has been illustrated that HCBP6 (FUNDC2), a mitochondrial outer membrane protein FUN14 structural domain containing 2, can interact with and destabilize SLC25A11, thereby affecting mGSH levels and regulating ferroptosis in the myocardium [61]. In addition, Wang et al., using organic proteomics and metabolomics approaches, identified a new mitochondrial transporter, SLC25A39, and highlighted its essential role in proliferation. Moreover, mice with systemic knockout of SLC25A39 (including the heart) exhibited severe anemia, a complete lack of red blood cells, and iron overload [62].

Mitochondrial membrane properties, controlled by the fatty acid composition and cholesterol/phospholipid molar ratio, also affect GSH transport. Compared to the plasma membrane, mitochondria have a lower cholesterol content. When the mitochondrial membrane is enriched in cholesterol, it decreases the activity of the GSH transporter system [57]. Previous studies in myocardial ischemia have revealed that ischemia leads to a progressive loss of cholesterol from tissues and the sarcoplasmic and sarcoplasmic reticulum, accompanied by a significant increase in mitochondrial cholesterol content that is admixed explicitly to this membrane system [63]. We thus hypothesize that myocardial mitochondrial membrane cholesterol enrichment due to ischemia may be one of the mechanisms of mGSH depletion in ischemic myocardium.

### Glutathione peroxidase

The glutathione peroxidase family comprises multiple isozymes (GPX1-8) existing in distinct subcellular locations and tissues. Among them, GPX1-4 and 6 are selenocysteine-containing proteins. Their active center is a conserved tetramer composed of selenocysteine residues (Sec), glutamine (Gln), tryptophan and asparagine. The active site of the other three GPXs is cysteine [64]. The presence of Sec as a catalytic group is thought to ensure a rapid reaction with hydrogen peroxide and a fast reduction of GSH [65]. Therefore, the most important function of GPX1-4 is to prevent oxidative stress by catalyzing the reduction of H_2_O_2_ or organic hydroperoxides. In addition, they can also inhibit inflammation and oxidant-induced programmed cell death (e.g., apoptosis, necroptosis, pyroptosis) [64, 66, 67]. GPX1 and GPX4 are the predominant antioxidant enzymes in the GPX family due to their widespread distribution. They are highly expressed in the cytoplasmic and mitochondrial compartments of cardiomyocytes and represent an important defense mechanism within the heart [4, 7]. GPX2 and GPX3 exert antioxidant effects in intestinal epithelial cells and plasma, respectively [65]. Compared with GPX1-3, GPX4 is also involved in the reduction of complex membrane-associated phospholipid hydroperoxides such as phosphatidylcholine hydroperoxide and cholesterol hydroperoxide, in addition to reacting with soluble, low molecular weight hydroperoxides such as t-butyl hydroperoxide and cumene hydroperoxide [68]. Since GPX4 has these characteristics, it plays a crucial role in inhibiting ferroptosis, which has received widespread attention. GPX5-8 without further ado due to the lack of antioxidant capacity.

## GSH system and ferroptosis in the myocardium

### Ferroptosis

Ferroptosis is a novel form of programmed cell death characterized by the accumulation of iron-catalyzed lipid peroxidation. It has its own unique morphological, biochemical, and genetic features. Morphological characteristics include reducing or disappearing mitochondrial cristae, small mitochondria with condensed mitochondrial membrane densities, and outer mitochondrial membrane rupture. Biochemically, once ferroptosis is activated, it cannot be suppressed by apoptosis, necrosis, or autophagy inhibitors but can be hidden by iron chelators such as deferoxamine (DFO) or antioxidants such as Trolox and fer-1. Moreover, the genetic aspects of the genetic network controlling ferroptosis differ from those controlling apoptosis [19]. As a new form of cell death, it provides new research directions for the treatment and thinking of many diseases. In addition, an increasing amount of research has supported a pathophysiological role for ferroptosis in the development of cardiovascular diseases, including myocardial ischemia‒reperfusion injury, myocardial infarction, adriamycin-mediated myocardial injury, and heart failure [69].

### The mechanisms of ferroptosis

Ferroptosis is a complex process regulated by various mechanisms. Its onset and execution involve three key events: iron accumulation, GSH depletion, and lipid membrane oxidation. The mechanisms associated with these events and their role in cardiovascular disease are outlined below.

#### Iron homeostasis

Regulation of intracellular iron homeostasis is influenced by iron uptake, utilization, storage, and export. Circulating iron uptake in the form of two ferric iron (Fe^3+^) bound to transferrin (TF) is achieved by binding to transferrin receptor protein 1 (TFR1) and subsequently triggering endocytosis [70]. Fe^3+^ absorbed into the cell is located in the endosomes and is reduced to ferrous iron (Fe^2+^) by transmembrane epidermal antigen of prostate 3 (STEAP3) [70]. Subsequently, through the action of divalent metal transporter 1 (DMT1), Fe^2+^ enters from the endosomes to the labile iron pool (LIP) in the cytoplasm [71]. A portion of the iron in the cytoplasm is transported to the mitochondria for the synthesis of heme and iron-sulfur clusters (ISCs) [72]. Part is stored in ferritin, which consists of ferritin heavy chain (FTH) and ferritin light chain (FTL) [72]. Iron is exported out of the cell via ferroportin (FPN), and it can oxidize Fe^2+^ to Fe^3+^ [73]. FPN is regulated by hepcidin, a hepatic-secreted peptide hormone, which promotes the internalization and degradation of FPN [74].

The augmentation of iron intake, the decline in stored iron, and the alleviation of iron outflow can lead to increased Fe^2+^ in the LIP in cells. Accumulated cellular iron generates cytotoxic hydroxyl radicals (OH·) via the Fenton reaction. It can attack polyunsaturated fatty acids (PUFAs) and produce lipid peroxides (lipid-OOHs) and their reactive degradation products [72, 75]. Cardiac-specific elision of Fth1 (encoding ferritin heavy chain) causes iron dysregulation and increased oxidative stress in the heart, leading to increased sensitivity to ferroptosis induced by iron overload [76]. Tang et al. showed that ubiquitin-specific protease 7 (USP7) led to increased TFR1 and ferroptosis in a rat model of myocardial ischemia/reperfusion [77]. TfR1 has been recognized as a specific target antigen associated with ferroptosis [78].

#### Lipid metabolism

Lipidomic studies have shown that phosphatidylethanolamines (PEs) with arachidonic acid (AA) or its derivative adrenergic acid (AdA) are the key phospholipids for oxidation, and their peroxidation is considered a significant driver of ferroptosis [19, 21]. Two key enzymes are implicated in PUFA-PE synthesis, including acyl-CoA synthetase long-chain family member 4 (ACSL4) and lysophosphatidylcholine acyltransferase 3 (LPCAT3) [75]. ACSL4 acylates AA, and subsequently, LPCAT3 catalyzes the acylated AA to membrane PL, which raises the oxidation of membrane-sensitive fatty acids such as PUFA, ultimately leading to lipid peroxidation [73]. Recently, it has been shown that overexpression of ACSL4 reverses the myocardial protective effect of baicalin on ischemia‒reperfusion and amplifies the degree of myocardial lipid peroxidation [79]. In addition to iron-catalyzed free radical chain reactions, lipoxygenase (LOXs) can directly oxidize PUFAs and lipid-containing PUFAs in cell membranes, which are considered to have an essential role in ferroptosis [75, 80]. One study reported that the scaffold protein phosphatidylethanolamine binding protein 1 (PEBP1) binds to 15-lipoxygenase (15LOX) and directs it to PUFAs in the membrane, thereby facilitating ferroptosis [81].

#### Antioxidant metabolism

The presence of the antioxidant system can prevent the infinite expansion of lipid peroxidation, which is a core step in impeding ferroptosis. As mentioned above, the GSH system has a vital role in the elimination of toxic lipid peroxides [32]; thus, it is essential for the inhibition of ferroptosis. Fang et al. selectively overexpressed SLC7A11 in the myocardium and found that it can increase cellular GSH levels and prevent FTH deficiency-mediated iron death [76]. Moreover, it has been previously shown that GPX4 overexpression in cancer cells inhibits RSL3-mediated ferroptosis, whereas GPX4 deletion increases susceptibility to ferroptosis [22]. These findings provide evidence for the importance of the GSH system in anti-ferroptosis.

Ferroptosis suppressor protein 1 (FSP1) was identified almost simultaneously by Bersuker et al. and Doll et al. as an anti-ferroptotic factor that parallels the GSH system [82, 83]. It can reduce ubiquinone (CoQ10) to ubiquinol (reduced form of CoQ10) in lipid membranes utilizing NADPH. The latter is a lipophilic radical scavenger that acts as an inhibitor of ferroptosis [82]. However, FSP1 must be myristoylated and recruited to lipid membranes to execute its reductase function [83]. In conclusion, FSP1, CoQ10, and NADPH comprise an anti-ferroptosis system parallel to GSH. In adriamycin-treated mouse hearts, elevated lipid peroxidation products promote FSP1 translocation [84]. Nevertheless, FSP1 has been less studied in the heart, where its relationship with ferroptosis and its precise function are poorly understood.

#### Other metabolic pathways

Glutamine is the most abundant and versatile amino acid in the body and provides a substrate for many biosynthetic processes [29]. Among them, the glutamate produced by the decomposition of glutamine via GLS not only provides the raw material for the synthesis of GSH but is also further metabolized into α-ketoglutarate (α-KG) in the mitochondria under the action of glutamate dehydrogenase (GLUD1) or transaminases and then enters the tricarboxylic acid cycle to produce ATP [29]. It has been reported that glutamine and glutaminolysis are required for cysteine starvation and erastin-mediated ferroptosis [85, 86]. Knockdown of GLS2 but not GLS1 can inhibit cysteine starvation-mediated ferroptosis of MEFs [86]. Notably, glutaminolysis inhibitors (compound 968) can prevent ischemia/reperfusion-induced cardiac damage [86]. However, it is unclear exactly which isoform acts in cardiomyocytes and how it exerts its anti-ferroptosis effect, which requires further study. Moreover, Gao et al. and Daiha Shin et al. found that exogenous α-KG mimics glutaminase-mediated ferroptosis, while using a transaminase inhibitor, amino-oxyacetate (AOA), reverses ferroptosis [85, 86]. This suggests that α-KG plays an essential role in glutaminolysis-mediated ferroptosis. Finally, knockdown of Gln transporters (SLC38A1 and SLC1A5) or pharmacological inhibition of SLC1A5 also significantly blocked cysteine starvation-mediated ferroptosis [85, 86].

### The GSH/GPX4 node is the crucial regulator of ferroptosis

The GSH system, as a lipid peroxide scavenger, has attracted much attention as a potential therapeutic target for ferroptosis. In the next section, we will discuss the relationship and importance between the GSH system and ferroptosis in myocardial injury.

#### Myocardial infarction (MI) or myocardial ischemia/reperfusion (MI/R) injury

Park et al. revealed that myocardial ferroptosis occurs during MI [87]. Subsequently, they used Western blot analysis and RNA sequencing (RNA-seq) to demonstrate that the expression of GPX4 was declining in the early (MI 1 day) and middle (MI 1 week) stages of MI. Inhibition or depletion of GPX4 utilizing the chemical inhibitor RSL3 or specific siRNA resulted in lipid peroxide accumulation, leading to ferroptosis in H9C2 cells [87]. These results suggest that the downregulation of GPX4 contributes to MI-induced ferroptosis. However, Tang et al. showed that GPX4, ACSl4, iron and malondialdehyde (MDA) in myocardial ischemic tissue did not change significantly after 15, 30 or 60 min of coronary artery occlusion [88]. The inconsistency of the above results may be because the duration of myocardial ischemia in the latter was too short to induce ferroptosis.

Currently, the primary treatment for myocardial infarction is opening blocked vessels to restore blood flow to the ischemic myocardium, which is called reperfusion. However, reperfusion can cause further damage to cardiac tissue, including cytokine production, neutrophil infiltration, and ROS production, called I/R injury [89]. Tang et al. continued reperfusion based on ischemia for 60 min. They found that iron, ACSL4, and MDA levels increased with increasing reperfusion time, with concomitant decreases in GPX4 levels, and deferoxamine treatment significantly ameliorated myocardial injury [88]. These results suggest that ferroptosis also occurs in myocardial ischemia‒reperfusion injury. In 2022, Lu et al. indicated that GPX4 and GSH levels were decreased, and cellular iron and MDA levels were increased in a mouse model of myocardial ischemia‒reperfusion (MIR, ischemia 30 min and reperfusion 24 h) injury [90]. Moreover, britanin, a bioactive terpenoid extracted from *Inula lineariifolia*, increased intracellular GPX4 and GSH levels and diminished ferroptosis-induced MIR injury via AMPK/GSK3β/Nrf2 signaling [90].

#### Doxorubicin (DOX)-induced cardiomyopathy (DIC)

A study by Fang et al. showed that the DOX-processing myocardium exhibited features of typical ferroptosis in mice. Ferroptosis inhibitors could significantly reduce DOX-induced cardiac injury and mortality. However, inhibitors of autophagy, necroptosis and apoptosis only partially improved survival substantially, suggesting that ferroptosis is essential for DOX-induced cardiomyopathy and mortality in mice [91]. Additionally, recent studies have found that the expression of GPX4 and GSH was disordered in rats administered DOX [92]. Fisetin, a natural flavonoid, attenuated DOX-mediated ferroptosis in vivo and in vitro by raising the contents of GSH and GPX4 and inhibiting the accumulation of MDA and iron in the heart of rats [92].

#### Septic cardiomyopathy (SIC)

In a model of LPS-mediated septic cardiomyopathy, some researchers revealed that LPS caused ferroptosis and septic cardiac injury by downregulating GPX4 protein levels and increasing COX-2 [93]. Knocking down ICA69 reversed GPX4 inhibition, attenuated intracellular ferroptosis production and improved mouse survival and cardiac function [93]. Furthermore, in appendiceal ligation and puncture-mediated sepsis, investigators found a decrease in GSH content and GPX4 expression and an accumulation of cardiac iron content and lipid peroxidation levels [94]. All of the above results suggest a possible involvement of ferroptosis in the development of sepsis-induced cardiac injury.

#### Diabetic cardiomyopathy (DCM)

Evidence has shown that ferroptosis is crucial for DCM pathogenesis [48]. Wang et al. elucidated that sulforaphane restored the downregulation of SLC7A11/GSH, suppressed ferroptosis, and improved cardiac function by activating the AMPK/Nrf2 pathway in the myocardium of type 2 diabetic mice [48]. Another study reported that the inhibition of lncRNA ZFAS1 upregulated cardiac GPX4 and terminated the ferroptosis process and oxidative insult in DCM mice [95].

#### Hypertension-mediated pathological cardiac remodeling

Zheng et al. used angiotensin (Ang) II injection to simulate a hypertension-mediated cardiac remodeling model and found that ferroptosis occurred and that the protein expression of GPX4 and xCT was downregulated in the myocardium. Elabela treatment reduced ferroptosis and improved myocardial hypertrophy and remodeling in hypertensive mice by normalizing the levels of xCT and GPX4 [96]. In addition, knockdown of xCT exacerbates Ang II-induced cardiac hypertrophy, fibrosis, and dysfunction in mice. These impairments can be relieved by hindering ferroptosis [97].

**Fig. 3:**
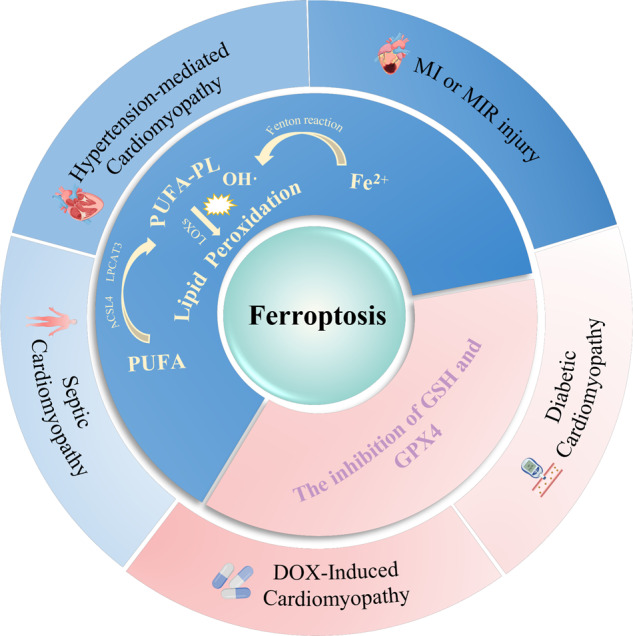
The main ferroptosis mechanisms in various types of cardiomyopathies. Among them, inhibition of the GSH system plays an important role in ferroptosis.

In summary, there is already substantial evidence that ferroptosis plays a vital role in myocardial injury caused by MIR and various types of cardiomyopathy. It has been proven feasible to treat heart disease by targeting ferroptosis modulators. GPX4 and GSH, as inhibitors of lipid peroxidation, are key regulators that control ferroptosis. In the myocardium affected by various pathological factors, the inhibition of GSH and GPX4 is ubiquitous. Therefore, activating GPX4 and GSH to inhibit ferroptosis is an attractive option for cardiac disease therapy (Fig. 3).

## Strategies for GSH system activation

Identifying strategies that fortify the GSH system in the heart may contribute to developing therapies against cardiac diseases induced by various pathological factors. This section summarizes potential approaches to increasing the GSH system in the heart.

### Improving the supply of raw materials for GSH systems

The direct administration of GSH in vivo is probably an obvious way to increase GSH It has been demonstrated that modified oral glutathione-like liposomal GSH increases erythrocyte and plasma GSH levels and reduces oxidative stress biomarkers [98]. Moreover, other routes of administration, such as intravenous, intranasal, and sublingual administration, can increase GSH levels [99]. However, the direct administration of GSH is rare in myocardial injury treatment studies.

In addition, supplementing the raw materials (cysteine, glycine, glutamate, and selenium) for synthesizing GSH or GPX to achieve an elevated GSH system in the myocardium is another feasible strategy. Next, we review the evidence supporting that increased raw material availability causes high GSH and GPX levels in the myocardium (Table 1, Supplementary Table S1).

**Table 1:** Clinical trials for the supply of raw materials for the GSH system.

Agent	Object	Dosage and duration	Treatment effect	Ref
NAC	Patients with AMI	15 g, IV over 24 h	Lowering plasma MDA concentration and GSSG/ GSH ratio, preserving better LV function.	[102]
	Patients with STEMI undergoing PCI	29 g, IV with 2 days	Reducing infarct size.	[103]
GlyNAC	Older adults	Gly 1.33 mmol/kg/day and NAC 0.81 mmol/kg/day, P.O for 24 weeks	Correcting GSH shortage of RBC, mitochondrial dysfunction and oxidative stress in RBCs, as well as ameliorating inflammation, insulin resistance, and endothelial dysfunction.	[110]
Glutamine	Patients undergoing cardiac surgery with extracorporeal CPB	0.5 g/kg/day, P.O for 3 days	Increasing plasma Gln concentrations and maintaining plasma GSH levels.	[113]

*NAC* N-acetylcysteine, *Gln* glutamine, *GSH* reduced glutathione, *GSSG* oxidized glutathione, *AMI* acute myocardial infarction, *MDA* malondialdehyde, *LV* left ventricle, *STEMI* ST elevation myocardial infarction, *PCI* percutaneous coronary intervention, *RBC* erythrocyte, *CPB* circulation cardiopulmonary bypass, *IV* intravenous injection, *P.O* oral.

#### Precursors of cysteine (Cys)

N-acetylcysteine (NAC) is a precursor of the amino acid cysteine. NAC deacetylates under the action of N-deacetylase to enhance intracellular Cys, thereby increasing the endogenous synthesis of GSH. In 2013, Rafeek HidhayathBasha et al. conducted a study on the protective effect of NAC on isoproterenol (ISO)-induced MI in rats [100]. The results demonstrated that pretreatment with NAC not only significantly increased the activities of GSH and GPX but also reduced the levels of lipid peroxidation in the heart mitochondria of ISO-treated rats [100]. Interestingly, NAC exerts a GSH-supplementing effect in GSH-deficient cells, whereas this effect may be ineffective in GSH-enriched cells [101].

In humans, clinical studies have evaluated the effects of NAC treatment on myocardial infarction. M A Arstall et al. found that the plasma MDA concentration and GSSG/GSH ratio were lower, and left ventricular function was better preserved in AMI patients treated with NAC (15 g, infusion over 24 hours) [102]. In addition, a recent study assessed the influence of intravenous high-dose NAC (29 g, IV within 2 days) combined with low-dose nitroglycerin (7.2 mg, IV within 2 days) on infarct size in patients with ST-elevation myocardial infarction undergoing percutaneous coronary intervention (PCI). The results indicated that patients treated with NAC had a 5.5% reduction in infarct size [103]. Therefore, NAC can be used to boost cardiac GSH levels and attenuate myocardial injury.

Apart from NAC, whey protein and methionine are also precursors of Cys. Whey protein and its concentrates are abundant in sulfur-containing amino acids such as Cys and methionine, so it can supplement cysteine and efficiently improve GSH levels [99, 104]. It has been reported that whey protein treatment added myocardial GSH/GPX content and decreased ROS in a mouse model of chronic iron overload cardiomyopathy [105]. Researchers do not recommend methionine because it causes an increase in homocysteine levels [99]. Elevated homocysteine is associated with various diseases, such as heart and brain diseases. Severe hyperhomocysteinemia in patients is able to develop neurological and cardiovascular disorders as well as premature death owing to complications [106]. A study revealed that L-methionine supplementation (1.7 g/kg/day, P.O for 8 weeks) was sufficient to produce hyperhomocysteinemia in rats and markedly increased the mean arterial pressure, oxidative stress and mean cardiomyocyte diameter [107].

#### Glycine

Glycine (Gly) is another essential amino acid in the synthesis of GSH. Although most studies have focused on increasing cysteine levels in vivo to boost GSH synthesis, glycine supplementation alone or in combination with NAC to increase GSH synthesis has also attracted extensive attention. In a burn rat model, intraperitoneal injection of glycine supplementation alleviated severe burn-induced cardiac injury by improving cardiomyocyte energy metabolism and increasing ATP and GSH contents [108]. Treatment of aged rat cardiac fibroblasts with NAC or NAC + Gly rose the intracellular GSH content, and notably, the combination of NAC and Gly exerted a better effect [109]. Premranjan Kumar et al. continuously fed a diet containing NAC + Gly to aged male mice for eight weeks, which corrected GSH levels and promoted the expression of GSH synthesis enzymes (GCLC, GCLM and GS) in the heart, liver and kidney, prolonging the lifespan of aged mice [50]. They also clarified in another clinical study that supplements with glycine (1.33 mmol/kg/day) and N-acetylcysteine (0.81 mmol/kg/day) for 24 weeks corrected erythrocyte-GSH deficiency and alleviated oxidative stress and mitochondrial dysfunction in elderly individuals [110]. Although the protective effect of glycine on MIR injury has also been investigated, whether it can augment the level of GSH in the ischemia‒reperfusion (I/R) myocardium is unclear.

#### Precursors of glutamate (Gln)

Gln acts as a precursor of glutamate (Glu), equivalent to being an indirect glutathione precursor. Its supplementation can enhance intracellular GSH levels and provide protection against myocardial injury. In ISO-induced myocardial infarction, pretreatment with Gln maintained cardiac GSH kurtosis and antioxidant enzyme (GPX, SOD, CAT) activity at near-normal levels, thus preventing oxidative damage in the myocardium [111]. A separate experiment elucidated that Gln supplementation increased cardiac GSH to halt myocardial injury induced by cyclophosphamide (CPA) in rats [112]. Notably, there is a significant correlation between plasma Gln concentrations and GSH levels. A double-blind, randomized, placebo-controlled trial demonstrated that perioperative high-dose intravenous injection of Gln (0.5 g/kg/day, P.O for 3 days) raised plasma Gln concentrations and maintained plasma GSH levels in postoperative patients undergoing cardiopulmonary bypass (CPB) [113].

#### Selenium

Selenium (Se) is an essential trace element for humans and animals, serving as the main component of many antioxidant proteins in the body, such as thioredoxin reductase, selenoprotein P and GPX. As a result, selenium may play an important role in protecting cardiomyocytes from oxidative stress [114]. Compared with a low-Se diet (0.05 mg/kg), a high-Se diet (1.5 mg/kg) maintained postischemic plasma GSH levels and GPX activity and improved myocardial infarct size and postischemic mean arterial blood pressure in rats [115]. Zhu et al. showed that Se boosted GPX1 expression by inhibiting DNMT2-mediated DNA methylation of the GPX1 promoter from lessening ROS production in advanced glycation end-product (AGE)-induced heart failure [114]. In humans, a systematic review and meta-analysis revealed that physiologically high selenium levels in the body were negatively associated with cardiovascular disease morbidity and mortality. When the blood selenium increment was within a specific range, the risk of CVD death decreased in a dose-dependent manner, but it seemed to start to increase when the increment exceeded 35 μg/L [116]. In addition, there is a U-shaped association between selenium exposure and some diseases, such as type 2 diabetes and fractures [117]. However, it has also been reported that selenium supplementation alone is not associated with cardiovascular disease and all-cause mortality [118].

### Glutathione system activator

Other than providing raw materials for the GSH system, another antioxidant strategy that activates the glutathione system is often achieved through medicinal therapy. In this section, we will describe their effects and mechanisms in detail by the source of the drugs.

#### Natural medicine monomers

Natural medicines have been a reliable source of drugs since ancient times, playing an essential role in preventing and treating various diseases. At present, increasing evidence has shown that compounds derived from natural medicines exert a variety of beneficial effects, including antioxidant, anti-inflammatory, and anti-apoptosis effects [119–121]. To date, more than 100 natural medicine monomers have been reported to activate the GSH system by several mechanisms to protect the heart from oxidative stress. Next, we will summarize some promising medicine monomers that activate the GSH system based on the categories of natural medicines, including flavonoids, terpenoids, phenolic acids, quinones, and alkaloids (Table 2, Supplementary Table S2).

**Table 2:** Clinical trials for natural medicine monomers.

Agent	Object	Dosage and duration	Treatment effect	Ref
Quercetin	Post-myocardial infarction patients	500 mg/day, P.O for 8 weeks	Improving serum TAC and reducing the insecurity scores.	[126]
	Patients with coronary heart disease	120 mg/day, P.O for 2 mouths	Improving cardiac function (both systolic and diastolic functions) and reducing the total duration and number of ST-segment depressions.	[127]
Genistein	Postmenopausal women with metabolic syndrome	54 mg/day, P.O for one year	Improving both cardiac functions and left atrial remodeling.	[138]
Soybean isoflavones(Genistein)	Patients with myocardial ischemia	80 mg/day, P.O for 24 weeks	Increasing the protein and mRNA levels of Nrf2 and the concentrations of SOD, and diminished serum levels of MAD, inflammatory factors.	[139]
β‐carotene	Patients with MI	–	Diminishing plasma lipid peroxide levels.	[146]
Lycopene	Patients with type 2 diabetes	10 mg/day, P.O for 2 months	Raising the ratio of serum TAC to MDA.	[149]
STS	Patients with non-STEMI receiving PCI	80 mg/day, IV for 2 days before and 3 days after PCI	Alleviating myocardial injury and the occurrence of short-term cardiovascular events.	[156]
Chlorogenic acid-enriched coffee	People with hypercholesterolaemia	–	Improving plasma antioxidant capacity and decreasing plasma lipid and protein oxidation as well as donating to enhancing cardiovascular health.	[169]
Ellagic acid	Patients with type 2 diabetic	180 mg/day, P.O for 8 weeks	Fortifying the mean of TAC as well as the activity of GPX enzymes and reducing MDA contents in the serum.	[172]
CoQ10	Patients with coronary artery diseases	300 mg/day, P.O for 12 weeks	Enhancing plasma GPX activity.	[178]
	Healthy adults	300 mg/day, P.O for 30 days	Increasing the GSH/GSSG ratio and reducing MDA concentration in the red blood cells.	[179]
Nigella sativa seed oil (Thy)	Hypertensive patients	–	Upregulating serum GR levels and diminishing serum MAD content, beneficial to glycemic and blood pressure control and lipid metabolism.	[181]
Curcumin	CHD patients	500 mg/day, P.O for 8 weeks 1000 mg/day, P.O for 12 weeks	Upregulating PPAR expression in red blood cells, boosting serum GSH and GPX levels and decreasing serum MAD contents.	[189, 190]
RSV	CHD patients with type 2 diabetes	500 mg/day, P.O for 4 weeks	Raising erythrocyte PPAR-γ and Sirt1 expression and serum TAC and attenuating the total/HDL cholesterol ratio.	[198]
	Patients with CHD	100 mg/day, P.O for 2 months	Improving LVEF and LV diastolic function in patients with CHD compared with standard treatment alone.	[199]

*P.O* oral, *TAC* total antioxidant capacity, *Nrf2* erythrocyte nuclear factor 2, *SOD* superoxide dismutase, *MDA* malondialdehyde, *IV* intravenous injection, *PPAR* peroxisome proliferator-activated receptor, *GR* glutathione reductase, *STS* sodium Tan IIA sulfonate injection, *CoQ10* coenzyme Q10, *Thy* thymoquinone, *CHD* coronary heart disease, *RSV* resveratrol, *LVEF* left ventricular ejection fraction, *STEMI* ST elevation myocardial infarction.

##### Flavonoids

Flavonoids are a group of phytochemicals widely existing in nature. They have received extensive attention for their antioxidant, anti-inflammatory, anti-mutagenic and anti-cancer properties, as well as their ability to protect against cardiovascular disease [122].

Quercetin (Quer) is one of the main flavonoids in many vegetables and fruits. Several preclinical studies have shown that Quer in the myocardium can enhance the GSH antioxidant system by regulating multiple upstream molecular targets, such as peroxisome proliferator-activated receptor-gamma (PPAR-γ), phosphatidylinositide 3-kinase (PI3K)/serine/threonine kinase (AKT), and Nrf2 [120, 123, 124]. It not only abrogates cardiac oxidant stress but also alleviates the inflammatory response and deterioration of heart function in MIR injury and DCM in rodents [123, 125]. In a recent randomized, double-blind, placebo-controlled clinical trial, Quer supplementation (500 mg/day, P.O for 8 weeks) evidently improved serum total antioxidant capacity (TAC) in postmyocardial infarction patients compared to the placebo group [126]. Furthermore, a clinical study from Natalia I Chekalina showed that adding Quer (120 mg/day, P.O for 2 mouths) to standard therapy improved cardiac function (both systolic and diastolic functions) and reduced the total duration and number of ST-segment depressions in patients with coronary heart disease (CHD) [127]. Thus, Quer, as an activator of the GSH system, is a promising natural small molecule for the treatment of cardiac injury.

Catechins, known as dihydroflavonols, are natural flavonoids present in green tea and other beverages, including catechin, epicatechin (EC), and epigallocatechin-3-gallate (EGCG) [122, 128]. It has been reported that these three compounds can enhance the contents of GSH and the activities of GSH-dependent antioxidant enzymes (GPX and GST) and reduce lipid peroxidation and cardiac damage mediated by DOX or isoproterenol (ISO) in the myocardium of rats, respectively [121, 129, 130]. Genistein is the main ingredient of isoflavone from soybean and is also used as a tyrosine kinase inhibitor. It has potent effects of antioxidant, anti-inflammatory, anti-angiogenesis, and anti-cancer [131]. Studies have shown that genistein can increase GSH levels and GPX activity along with decreased oxidative stress in diabetic cardiomyopathy and DOX-mediated cardiomyopathy [132, 133]. Meanwhile, genistein increased the levels of Nrf2, which is vital to the GSH system [132–134]. Genistein administration induced Nrf2 enhancement by activation/phosphorylation of keap1, sirt1, MAPK-ERK1/2, PKC, and estrogen receptor in other cells or tissues, such as: hippocampal tissue, hypothalamic paraventricular nucleus, ovary, and Caco-2 cells [131, 135–137]. However, it is unclear why genistein upregulates the levels of Nrf2 in the myocardium. In a clinical trial, pure genistein supplementation (54 mg/day, P.O for one year) improved both cardiac function and left atrial remodeling in postmenopausal women with metabolic syndrome [138]. Another randomized controlled trial showed that soybean isoflavone therapy (80 mg/day, P.O for 24 weeks) significantly increased the protein and mRNA levels of Nrf2 and the concentrations of SOD as well as diminished serum levels of inflammatory factors and MAD in patients with myocardial ischemia [139]. Therefore, genistein, an antioxidant, might be a potential drug for treating patients with myocardial injury.

##### Terpenoids

Terpenoids are natural compounds with a wide distribution, great variety, and diversified structure in nature [140]. Terpenoids have been reported to activate the GSH antioxidant system to protect the heart from oxidative stress and alleviate cardiac injury.

Tetraterpenes, also called carotenoids, are widespread in higher plants, fungi, algae, and animals and are one of the most potent natural antioxidants [141]. Among them, β‐carotene is one of the most frequently available dietary carotenoids for human consumption [142]. In preclinical studies, β‐carotene tended to maintain myocardial GSH levels and GPX activities and reduce oxidative stress in vitro and in vivo [143, 144]. It could also alleviate the size of myocardial infarction and the subsequent decrease in cardiac function in rats [145]. A clinical study of patients with myocardial infarction by B Panczenko-Kresowska et al. revealed that β-carotene supplementation remarkably diminished plasma lipid peroxide levels in patients [146]. The mechanism might be related to its own antioxidant properties and GSH activating effect. Nonetheless, the most updated literature reports that β-carotene supplementation or elevated blood β-carotene levels may increase all-cause mortality and CVD risk [147, 148]. Moreover, a natural compound of the tetraterpene group from tomatoes, lycopene (10 mg/day, P.O for 2 months), was reported to significantly increase the ratio of serum TAC to MDA in patients with type 2 diabetes [149]. Considering that this compound has been demonstrated to expand the GSH system along with a reduction in oxidative injury by the AKT/Nrf2 signaling pathway in the hearts of rats and H9C2 cells, the abovementioned enhanced TAC may cover GSH and GSH-dependent antioxidant enzymes [150–152].

In addition, tanshinone IIA (Tan IIA) is a diterpene extracted from the root and rhizome of *Salvia miltiorrhiza Bunge*. Its water-soluble derivative, sodium Tan IIA sulfonate injection (STS), is widely used in the clinic as an adjuvant drug for coronary heart disease, myocardial infarction and heart failure in China [153]. In an I/R-mediated myocardial injury model, pretreatment with Tan IIA activated the GSH system by increasing Nrf2 expression [154]. Tan IIA induced activation of the GSH system to attenuate cardiac oxidative stress and restrain the increase in serum myocardial enzymes, thus relieving the extent of myocardial damage [154, 155]. As an aside, in a randomized, double-blind, placebo-controlled study, the addition of STS (80 mg/day, IV for 2 days before and 3 days after PCI) to the standard treatments could alleviate myocardial injury and the occurrence of short-term cardiovascular events in patients with non-ST elevation acute coronary receiving PCI [156]. Moreover, a meta-analysis showed that the addition of STS seemed more effective in treating heart failure than Western medicine alone [153]. The excellent clinical manifestations of Tan IIA provide supportive evidence for targeting the GSH system in the treatment of heart diseases, which may be promising for transforming natural compounds from basic research to clinical applications.

##### Phenolic acid

Phenolic acids are attracting increasing attention for their antioxidant properties and other health benefits. Based on differences in the carbon skeleton, phenolic acids can be mainly divided into two categories: hydroxycinnamic acid (e.g., cinnamic acid, ferulic acid, caffeic acid, and chlorogenic acid) and hydroxybenzoic acid (e.g., gallic acid, ellagic acid, syringic acid, and Danshensu) [157]. All of the compounds above have been proven to maintain GSH contents and GSH-dependent antioxidant enzyme activities in the myocardium, protecting it from oxidative stress [41, 158–164].

Ferulic acid (FA), the main active component isolated from *Angelica sinensis*, has been documented to increase the levels of GSH and the expression of GPX in the myocardium by upregulating AMPKα2 and activating Nrf2 signaling [41, 165]. Moreover, FA-induced enhancement of the GSH system attenuated lipid peroxidation and prevented cardiac ferroptosis mediated by I/R [41]. In recent years, ferulic acid derivatives have been widely investigated. Of them, sodium ferulate (SF) features low toxicity, stability, ease of synthesis, and water solubility and has been authorized by the State Drug Administration of China as an adjuvant drug for treating ischemic cardiovascular diseases [166, 167]. It could also increase GPX activity, thereby exerting antioxidative effects in rats [166].

Besides, caffeic acid and chlorogenic acid (caffeoylquinic acids) are two other classical phenolic acids widely found in natural plants, both of which are the most vital active substances in coffee [168]. Some investigations have demonstrated that the two compounds, acting as agonists of the GSH antioxidant system, well prevent oxidative stress and reduce heart damage in some animal cardiomyopathy models, for instance, the ISO-induced myocardium infarction model [159, 160]. Additionally, one clinical study reported that consumption of chlorogenic acid-enriched coffee not only improved plasma antioxidant capacity and decreased plasma lipid and protein oxidation but also donated to enhancing cardiovascular health in people with hypercholesterolemia [169]. Interestingly, Yuki Sato and coworkers indicated that the antioxidant effect of caffeic acid is more potent than chlorogenic acid, and the latter is metabolized into caffeic acid in the intestine. Therefore, they speculated that caffeic acid probably acts as a major player in the preventive effect of chlorogenic acid against oxidative damage in ischemia‒reperfusion intestinal tissue [170].

Ellagic acid (EA) also increased the GSH antioxidant system and diminished oxidative stress in several models of myocardial injury, such as diabetes and cisplatin-mediated heart injuries [164, 171]. In humans, EA intervention (180 mg/day, P.O for 8 weeks) significantly fortified the mean of TAC and the activity of GPX enzymes as well as reduced MDA contents in the serum of type 2 diabetic patients [172]. Nonetheless, it is not certain whether EA may be helpful in boosting the GSH system in cardiac patients and treating heart diseases. Thus, there is a need for larger studies to fully address this issue. In addition, EA was found to have poor low oral bioavailability, thus limiting its clinical applications. In contrast, its intestinal microbial metabolite, urolithin, possesses better biological activity and higher bioavailability [173]. It has been reported that urolithin A also increased total GSH, lowered oxidative stress, and improved cardiac function in the myocardium of diabetic rats [174].

##### Quinones

Coenzyme Q10 (CoQ10) is a fat-soluble quinone. It is also a natural antioxidant molecule in the body. The positive effect of CoQ10 treatments is recognized in patients with heart failure and myocardial ischemia, such as the reduction of oxidative stress derived from cardiovascular causes, decrease in mortality and hospitalization and improvement in cardiac function [175, 176]. Thereinto, the antioxidant effect of CoQ10 may be partially attributed to activating the GSH system. In a rat model of ISO-mediated myocardial infarction, CoQ10 preconditioning not only markedly expanded cardiac GSH levels and reduced the concentration of lipid peroxidation but also alleviated myocardial damage [177]. In humans, a randomized controlled clinical trial evaluated the effect of CoQ10 administration (300 mg/day, P.O for 12 weeks) on the antioxidant system in patients with coronary artery diseases. The findings revealed that plasma GPX activity was significantly increased after CoQ10 therapy and was positively related to plasma coenzyme Q10 levels [178]. Moreover, CoQ10 treatment (300 mg/d, P.O for 30 days) induced a significant increase in the GSH/GSSG ratio and a remarkable reduction in MDA concentration in the red blood cells of healthy adults [179].

Thymoquinone (Thy), a benzoquinone compound, is the main active ingredient in the extract of Nigella sativa seeds. Its treatment was reported to increase the levels of GSH and the activities of GPX and GST, along with a decrease in cardiac lipid peroxide production in a rat model of myocardial injury caused by diazinon (a pesticide) [180]. Moreover, Thy-rich Nigella sativa seed oil exerts an antioxidant effect as an adjuvant therapy in hypertensive patients, mainly manifested in the upregulation of serum glutathione reductase (GR) levels and reduction of serum MAD content. It also showed beneficial impacts on glycemic and blood pressure control as well as lipid metabolism in hypertensive patients without serious adverse effects such as liver and kidney dysfunction [181]. In addition, some quinone compounds, such as β-LAPachone and aloin, have similar effects on heart protection [182, 183]. We will not give unnecessary details here.

##### Others

In addition to the above compounds, many other natural medicines potentially protect the myocardium by enhancing the GSH antioxidant system. Curcumin is a classic curcuminoid extract from the rootstock of turmeric. Numerous preclinical reports have highlighted the ability of curcumin to restore reduced GSH levels and the activities of GSH-related antioxidant enzymes in the myocardium after exposure to cardiotoxic factors, for example, DOX, I/R, diabetes, hyperthyroid conditions, and copper sulfate [184–188]. Upstream molecular targets, such as Nrf2, Sirt3, and PPAR-γ, are responsible for the curcumin-enhanced GSH system [184–186]. Moreover, curcumin treatment not only quells oxidative stress in the myocardium but also impedes myocardial inflammation, which improves heart function and decreases heart damage [184–188]. Two recent clinical studies also reported remarkably upregulated PPAR expression in red blood cells, boosted serum GSH and GPX levels and decreased serum MAD contents after treatment with curcumin (500 mg/day, P.O for 8 weeks; 1000 mg/day, P.O for 12 weeks) in patients with CHD [189, 190]. Considering that curcumin is nontoxic, readily available and inexpensive, it is a prospective candidate for treating heart diseases.

Resveratrol (RSV) is a stilbene found in grapes, wine and blueberries that possesses potent antioxidant activity to protect the myocardium from oxidative damage. In particular, the enhanced GSH system plays an essential role in the antioxidant capacity of RSV [191]. In 2009, Elif Tatlidede et al. demonstrated that RSV treatment notably raised cardiac GSH contents and suppressed oxidant responses in DOX-processed rats; Simultaneously, it ameliorated DOX-induced deterioration of cardiac function and myocardial injury [192]. In the following decade, many researchers verified in various models of myocardial injury that RSV can exert an antioxidant effect and protect the myocardium by increasing the level of GSH and upregulating the activity of GSH-dependent antioxidant enzymes [193–197]. Moreover, they revealed that the mechanism by which RSV enhances the GSH system involves the AMPK signaling pathway, the Nrf2 signaling pathway, the Sirt1 pathway, noncoding RNA (miR-149) and the KAT5 gene [193–197]. In addition, RSV can suppress the occurrence of ferroptosis in the myocardium by increasing SLC7A11/GSH and GPX4 [193, 195]. In general, consistent with preclinical studies, RSV supplementation (500 mg/day, P.O for 4 weeks) raised erythrocyte PPAR-γ and Sirt1 expression and serum TAC and attenuated the total/HDL cholesterol ratio in CHD patients with type 2 diabetes [198]. Another clinical study clarified that adding RSV (100 mg/day, P.O for 2 months) better improved left ventricular ejection fraction and left ventricular diastolic function in patients with CHD compared with standard treatment alone [199].

#### Synthetic medicines

Synthetic drugs have been researched and developed in recent modern times, a proportion of which have already been in clinical use for the treatment of various diseases, such as myocardial infarction, heart failure, diabetes, hypertension, oncology, and sepsis. Similar to natural drugs, many synthetic drugs have properties that enhance the GSH system and thus protect cells from oxidative damage. The following section describes and summarizes these drugs in terms of the mechanisms by which they activate the GSH system (Table 3, Supplementary Table S3).

**Table 3:** Clinical trials for synthetic medicines.

Agent	Object	Dosage and duration	Treatment effect	Ref
Melatonin	Patients undergoing CABG	10 mg/day, P.O for 30 days	Raising Nrf2 levels in peripheral blood mononuclear cells.	[208]
	CHD patients with type 2 diabetic	10 mg/day, P.O for 12 weeks	Increasing serum GSH levels while reducing serum MDA and protein carbonyl (PCO) in CHD patients with type 2 diabetes. as well as beneficial effecting on patients’ glycemic and blood pressure control, serum hs-CRP levels, total cholesterol/HDL cholesterol ratio, total cholesterol, and mental health parameters.	[209]
Pioglitazone	Diabetic patient	30 mg/day, P.O for 4 weeks	Decreasing in MDA.	[218, 219]
	Patients with cardiovascular diseases (CVD) and diabetic patients who had previous myocardial infarction	–	Reducing the risk of MI and acute coronary syndrome. However, increasing the risk of the development of heart failure.	[220–222]
Metformin	CHD patients without diabetes	2000 mg/day, P.O for 12 months	Declining oxidative stress, lowering LVM, and improving blood pressure.	[227]
Atorvastatin	CHF patients	20 mg/day, P.O for 4 weeks	Reducing plasma markers of oxidative stress (MDA) while improving the functional capacity assessed by 6MWT.	[228]
DEX	Patients undergoing cardiac valve replacement	0.5 μg/kg/h, pumping injection, before induction of anesthesia to the end of surgery	Reducing cardiac troponin I and MDA levels and decreasing the incidence of arrhythmias.	[234, 235]
	Patients who underwent cardiac surgery	0.24 to 0.6 μg/kg/h, IV, after cardiopulmonary bypass and continued for <24 h postoperatively	Reducing postoperative in-hospital, 30-day and 1-year mortality.	[236]
Captopril	Type 2 diabetes patients	12.5 mg/day, P.O for 3 months	Improving the reduced plasma level of GSH.	[241]
Probucol	CHD patients undergoing PCI	500 mg twice daily, IV one day before and three days after surgery	Increasing serum GSH levels.	[244]
	Patients with CHD	500 mg/day, P.O for 6 months	Lowering the incidence of the patient’s primary endpoint (cardiovascular disease death, hospitalization rate).	[245]

*PCO* protein carbonyl, *CABG* coronary artery bypass grafting, *CHF* chronic heart failure, *6MWT* 6-minute walk test, *DEX* dexmedetomidine, *hs-CRP* high-sensitivity c-reactive protein.

##### Activating upstream targets of the GSH system

The key upstream target for GSH system modulation is generally considered to be Nrf2, a redox-sensitive regulator. Nrf2 is involved in not only the regulation of GSH synthesis and reduction via upgrading some enzymes and proteins (SLC7A11, GCLM, GCLC, GR) but also the activation of GSH-dependent antioxidant enzymes (GPX, GST) [200, 201]. Furthermore, Gobinath Shanmugam et al. revealed that in an ISO-mediated model of myocardial infarction, direct overexpression of the Nrf2 gene enhanced GSH levels in cardiomyocytes and guarded cardiomyocytes against oxidative damage and lessened the occurrence of ferroptosis [202]. This further confirms that the enhancement of Nrf2 is a viable strategy for activating the GSH system.

The representative synthetic compound targeting Nrf2 is melatonin, an amine hormone secreted by the brain’s pineal gland [203]. Melatonin prevents Nrf2 degradation and augments its nuclear accumulation by inhibiting proteasomal [204]. In in vivo and in vitro studies, melatonin was proven to increase the level of Nrf2 in cardiomyocytes exposed to oxidative stress. Based on this, in the subsequent I/R- and TMT-induced myocardial injury model, the drug not only enhanced GSH levels and GPX and GST activities to lower oxidative damage but also reduced TMT-induced pyroptosis and I/R-mediated apoptosis [205–207]. Among them, Cai et al. revealed the grievous connection between oxidative stress, pyroptosis, inflammation response, and xenobiotic metabolism in the myocardium of TMT-treated rats by String database analyzing the proteome interaction protein [206]. In addition, Shaghayegh Haghjooy Javanmard et al. indicated that preoperative treatment with melatonin (10 mg/day, P.O for 30 days) notably raised Nrf2 levels in peripheral blood mononuclear cells of patients undergoing coronary artery bypass grafting (CABG) [208]. In 2017, another clinical study showed that melatonin intake (10 mg/day, P.O for 12 weeks) increased serum GSH levels while reducing serum MDA and protein carbonyl (PCO) in CHD patients with type 2 diabetes. Additionally, it has beneficial effects on patients’ glycemic and blood pressure control, serum high-sensitivity C-reactive protein (hs-CRP) levels, total cholesterol/HDL cholesterol ratio, total cholesterol, and mental health parameters [209].

Additionally, it was reported that trimetazidine (TMZ), a piperazine derivative, mediated activation of the GSH system and had antioxidant and cytoprotective effects in several myocardial injury models [210–213]. For instance, in the myocardium of exhaustive-exercised rats, TMZ produced specific cardioprotective outcomes by increasing GSH and GPX activity to clear oxidative stress. Further mechanistic studies have found that treatment with TMZ promoted cardiac Nrf2 expression, indicating that TMZ enhanced the clearance of oxidative stress by activating the GSH system through the Nrf2 signaling pathway [210]. Moreover, TMZ also has other benefits to the myocardium, including increased coronary blood flow reserve, maintenance of cardiac energy metabolism, anti-apoptosis, and anti-inflammation. It has been approved for clinical use in the therapy of angina pectoris [210, 211].

In addition to Nrf2, activation of PPAR-γ and the AMPK pathway can also facilitate the GSH system. Rosiglitazone and pioglitazone belong to the thiazolidinedione class of hypoglycemic agents and are recognized as PPAR-γ agonists. These two drugs were not only able to enrich GSH contents and GPX activity and defend the heart against oxidative stress but also prevented DOX or I/R-induced myocardial injury [214–217]. Some clinical studies have shown that pioglitazone treatment (30 mg/day, P.O for 4 weeks) exhibits antioxidant properties in diabetic patients, as reflected by a decrease in MDA [218, 219]. In addition, there is evidence that pioglitazone reduces the risk of MI and acute coronary syndrome in patients with cardiovascular diseases (CVD) and patients with type 2 diabetes who had previous myocardial infarction [220–222]. Notably, it increases the risk for the development of heart failure [221, 222]. Compared to pioglitazone treatment, rosiglitazone increases the risk of AMI and heart failure in type 2 diabetic patients and elderly patients [222–224]. Metformin is a traditional drug for the treatment of type 2 diabetes. In addition to its antidiabetic results, it was also found to achieve cardiac protection by activating the AMPK pathway [225]. Some researchers have shown that metformin activates AMPK and triggers the downstream GSH system to slow DOX-mediated oxidative impairment in the myocardium [225, 226]. A randomized controlled trial on the effect of metformin in CHD patients without diabetes showed that it (2000 mg/day, P.O for 12 months) not only decreased oxidative stress but also lowered left ventricular mass (LVM) and improved blood pressure [227].

##### Enhancing GSH synthesis

SLC7A11 is an essential molecule that limits the Cys transport rate, thus restricting the synthesis of GSH. Direct SlC7A11 overexpression in cardiac myocytes boosted GSH levels and reduced cardiac ferroptosis and injury, suggesting that it is feasible to fortify GSH levels by increasing SLC7A11 [76].

Atorvastatin, a statin that reduces cholesterol, is a first-line drug for treating cardiovascular disease. It was reported that it could raise intracellular GSH by increasing SLC7A11 expression, efficiently leading to GPX4 enhancement and ferroptosis suppression in the ISO-induced injury model of H9C2 cells and rat myocardium [49]. Additionally, one clinical trial by Douglas Greig et al. revealed that adding atorvastatin (20 mg/day, P.O for 4 weeks) reduced plasma MDA while improving the functional capacity assessed via a 6-minute walk test in patients with chronic heart failure (CHD) [228]. It is important to note that a recent study revealed the opposite result with high doses of atorvastatin (40 µm) in human cardiomyocytes and murine skeletal muscle cells, which is consistent with the adverse muscle effects of atorvastatin [229]. Other statin drugs, such as rosuvastatin and fluvastatin, can increase GSH and/or GPX in the myocardium to alleviate ISO-mediated myocardial injury [230, 231]. However, it is not clear whether they regulate the expression of SLC7A11.

Dexmedetomidine (DEX), an α2-adrenergic receptor, also augments the expression of SLC7A11. In 2022, one study discovered that DEX postconditioning could enhance GSH and GPX4 levels in the myocardium, preventing cardiac damage and ferroptosis caused by I/R [232]. In the same year, Wang et al. showed that DEX increased the expression levels of SLC7A11 and GPX4 and protected H9C2 cells from hypoxia/reoxygenation injury through the AMPK/GSK-3β/Nrf2 axis [233]. Moreover, DEX treatment (0.5 μg/kg/h, pumping injection, before induction of anesthesia to the end of surgery) has been reported to reduce cardiac troponin I and MDA levels and to decrease the incidence of arrhythmias in patients undergoing cardiac valve replacement [234, 235]. A previous clinical study also showed that perioperative DEX (0.24 to 0.6 μg/kg/h, IV, after cardiopulmonary bypass and continued for <24 hours postoperatively) reduced postoperative in-hospital, 30-day and 1-year mortality in patients who underwent cardiac surgery [236]. These results suggest that DEX has antioxidant and cardioprotective potential. However, clinical studies of the drug have focused on surgical patients. It is unclear whether it exhibits cardioprotective effects in patients with post-PCI, AMI, or other cardiomyopathies.

## Increasing GSH and/or GSH-dependent antioxidant enzymes directly

Many studies have concentrated on the direct effects of synthetic drugs on GSH and GSH-dependent antioxidant enzymes in damaged myocardium without further mechanistic investigations. Herein, we discuss some critical examples of representative synthetic compounds.

Captopril, an angiotensin-converting enzyme inhibitor, is widely used in clinical practice to treat hypertension, MI, and congestive heart failure and is effective in attenuating left ventricular (LV) dilatation, ameliorating LV ejection fraction and improving cardiovascular morbidity and mortality [237, 238]. Captopril has been revealed to facilitate the GSH system in several rodent models of myocardial injuries, such as clozapine and DOX-induced damage models, thereby protecting cardiomyocytes from oxidative stress [239, 240]. A recent clinical trial in type 2 diabetes patients with CVDs following captopril treatment (12.5 mg/day, P.O for 3 months) found a significant improvement in plasma GSH levels and a concomitant decline in lipid peroxidation [241]. Consequently, these results partially explain the pharmacological mechanism of captopril as a cardioprotective agent.

Similar to the statin lipid-lowering drugs mentioned earlier, other lipid-lowering drugs, such as probucol, also exhibit the capability to restore the inhibited GSH system and defend the myocardium from oxidative damage after exposure to pathological factors, for example, ISO and cyclophosphamide [242, 243]. More recently, in CHD patients undergoing PCI, intravenous administration of probucol (500 mg twice daily, one day before and three days after surgery) increased serum GSH levels [244]. Moreover, using probucol (500 mg/day, P.O for 6 months) for secondary prevention in patients with CHD lowered the incidence of the patient’s primary endpoint (cardiovascular disease death, hospitalization rate), which may be partly due to its strong antioxidant capacity [245].

### Others

Some nondrug treatment modalities, such as exosome and gene therapy, have been documented to regulate the myocardium’s GSH system. Exosomes are lipid membrane nanovesicles 40–100 nm in diameter [246]. Exosomes (EXOS) of mesenchymal stem cells (MSCs) from human umbilical cord blood increased GSH and attenuated lipid peroxidation and ferroptosis in cardiomyocytes in a rat infarction model. However, it has no effect on the level of GPX4 [247]. A recent study reported that exosomes derived from mouse bone marrow MSCs exerted similar cardio-protective effects [248]. However, not all exosomes positively impact the myocardium and GSH. For instance, exosomes secreted by adipose tissue macrophages caused ferroptosis by targeting SLC7A11 to inhibit GSH synthesis in the heart [249].

Current gene therapy research aims to target, for example, upstream targets of the GSH system or molecules associated with it (such as Nrf2, GPX, SLC7A11), genes aberrantly expressed in myocardial injury, and noncoding RNAs regulating mRNA transcription [76, 202, 250–253]. Recombinant human GPX4 alleviated ISO-induced myocardial ischemia injury [250]. SLC7A11 overexpression boosted cardiac GSH levels and reduced cardiac ferroptosis and injury in cardiomyopathy caused by loss of cardiac ferritin H (Fth^MCK/MCK^) [76]. In addition, some investigators found that USP22 and lncRNA PART1 expression was decreased in a MIR injury model. Rodents overexpressing USP22 or lncRNA PART1 were well resistant to I/R-induced oxidative stress and cardiac injury, which may be partly attributed to the elevated GSH content in the myocardium [251, 252]. In particular, USP22 raised GSH content and reduced the occurrence of ferroptosis in cardiomyocytes by activating the Sirt1-p53/SLC7A11 axis [252]. In addition, Zhang et al. overexpressed miR-340-5p in the myocardium by direct myocardial injection of AVV-9 containing miR-340-5p precursor, which targeted inhibition of MyD88 and thus restored declining GSH levels, alleviated insult of cardiac function and oxidative injury induced by sepsis in the hearts of mice [253].

## Conclusions and perspective

The GSH system is one of the most significant members of the cellular antioxidant defensive system, which is essential for eliminating excess ROS and protecting the myocardium in the presence of pathological cardiovascular factors. Notably, glutathione balance and GPX4 levels are deeply involved in the susceptibility of cardiomyocytes to ferroptosis, a novel form of cell death. Furthermore, many researchers have found that reduced GSH levels and GSH-dependent antioxidant enzyme activity in damaged myocardium due to excess ROS and disruption of the GSH synthesis process, along with increased oxidative damage and ferroptosis. Based on this, therapeutic strategies to restore the activity of the GSH system in the heart are beneficial in the treatment of myocardial injury. Precursors of GSH or GPX biosynthesis, various small molecule activators related to the GSH system and some novel therapeutic approaches, such as exosomes and gene therapy, can boost the activity of the GSH system and reduce cardiac damage and ferroptosis.

Notably, despite constant progress in diverse aspects of natural drug monomers that protect against myocardial injury by activating the GSH system, great challenges remain in translating these compounds into future clinical pharmaceuticals. First, most natural small molecules present low bioavailability problems [254], so further research on delivery systems and formulations for these drugs is needed. Second, the development of new drugs requires additional and more detailed preclinical and clinical studies to determine whether long-term use is biotoxic, whether the drug has adverse effects on other peripheral organs or whether the drug candidate has a real therapeutic effect on the patients.

In addition, a portion of synthetic agents have been used clinically to treat various diseases, such as myocardial infarction, heart failure, diabetes, hypertension, tumors, and sepsis. These studies on myocardial injury and the GSH system reveal new roles of these clinical drugs in the treatment and further elaborate their mechanisms, which provide new ideas for their clinical application. However, more clinical studies must be encouraged to evaluate whether these drugs can act as GSH system enhancers and cardioprotective agents in patients.

Moreover, some emerging therapeutic approaches to enhance the GSH system have attracted much attention. Currently, there are clinical studies using AAV vectors to transfect sarcoplasmic/endoplasmic reticulum Ca (2+)-ATPase (SERCA2a) to treat heart failure [255, 256]. These results show that although the expected therapeutic effect has not been achieved, AVV-based therapy has acceptable safety. This opens up the possibility of our gene therapy targeting the GSH system.

Overall, we can see the value of targeting the GSH system in myocardial injury. Despite being faced with many problems, it provides new directions and ideas for the treatment of myocardial injury. The continuous exploration of new treatment modalities will be critical to successfully combat myocardial damage in the future.

## Supplementary information

Supplementary Material

checklist

## Data Availability

All data generated or analyzed during this study are included in this published article and its supplementary information files.

## References

[1] Roth GA, Mensah GA, Johnson CO, Addolorato G, Ammirati E, Baddour LM (2020). Global burden of cardiovascular diseases and risk factors, 1990-2019: update from the GBD 2019 study. J Am Coll Cardiol.

[2] Kong X, Liu H, He X, Sun Y, Ge W (2020). Unraveling the mystery of cold stress-induced myocardial injury. Front Physiol.

[3] Ansley DM, Wang B (2013). Oxidative stress and myocardial injury in the diabetic heart. J Pathol.

[4] D’Oria R, Schipani R, Leonardini A, Natalicchio A, Perrini S, Cignarelli A (2020). The role of oxidative stress in cardiac disease: from physiological response to injury factor. Oxid Med Cell Longev.

[5] Raedschelders K, Ansley DM, Chen DDY (2012). The cellular and molecular origin of reactive oxygen species generation during myocardial ischemia and reperfusion. Pharmacol Therapeutics.

[6] Zhang J, Pan W, Zhang Y, Tan M, Yin Y, Li Y (2022). Comprehensive overview of Nrf2-related epigenetic regulations involved in ischemia-reperfusion injury. Theranostics..

[7] Dubois-Deruy E, Peugnet V, Turkieh A, Pinet F. Oxidative stress in cardiovascular diseases. Antioxidants. 2020;9:864. 10.3390/antiox9090864 PMC7554855 32937950

[8] van der Pol A, van Gilst WH, Voors AA, van der Meer P (2019). Treating oxidative stress in heart failure: past, present and future. Eur J Heart Fail.

[9] Sawyer DB, Colucci WS (2000). Mitochondrial oxidative stress in heart failure: “oxygen wastage” revisited. Circ Res.

[10] Perrelli M-G, Pagliaro P, Penna C (2011). Ischemia/reperfusion injury and cardioprotective mechanisms: Role of mitochondria and reactive oxygen species. World J Cardiol.

[11] He L, He T, Farrar S, Ji L, Liu T, Ma X (2017). Antioxidants maintain cellular redox homeostasis by elimination of reactive oxygen species. Cell Physiol Biochem.

[12] Matuz-Mares D, Riveros-Rosas H, Vilchis-Landeros MM, Vazquez-Meza H. Glutathione participation in the prevention of cardiovascular diseases. Antioxidants. 2021;10:1220. 10.3390/antiox10081220 PMC8389000 34439468

[13] Forman HJ, Zhang H, Rinna A (2009). Glutathione: overview of its protective roles, measurement, and biosynthesis. Mol Asp Med.

[14] Jaganjac M, Milkovic L, Sunjic SB, Zarkovic N. The NRF2, thioredoxin, and glutathione system in tumorigenesis and anticancer therapies. Antioxidants. 2020;9:1151. 10.3390/antiox9111151 PMC7699519 33228209

[15] Barth E, Stammler G, Speiser B, Schaper J (1992). Ultrastructural quantitation of mitochondria and myofilaments in cardiac muscle from 10 different animal species including man. J Mol Cell Cardiol.

[16] Brown DA, Perry JB, Allen ME, Sabbah HN, Stauffer BL, Shaikh SR (2017). Expert consensus document: Mitochondrial function as a therapeutic target in heart failure. Nat Rev Cardiol.

[17] Chen Y, Saari JT, Kang YJ (1994). Weak antioxidant defenses make the heart a target for damage in copper-deficient rats. Free Radic Bio Med.

[18] Franco R, Cidlowski JA (2012). Glutathione efflux and cell death. Antioxid Redox Signal.

[19] Dixon SJ, Lemberg KM, Lamprecht MR, Skouta R, Zaitsev EM, Gleason CE (2012). Ferroptosis: an iron-dependent form of nonapoptotic cell death. Cell..

[20] Wu X, Li Y, Zhang S, Zhou X (2021). Ferroptosis as a novel therapeutic target for cardiovascular disease. Theranostics..

[21] Stockwell BR, Friedmann Angeli JP, Bayir H, Bush AI, Conrad M, Dixon SJ (2017). Ferroptosis: a regulated cell death nexus linking metabolism, redox biology, and disease. Cell..

[22] Yang WS, SriRamaratnam R, Welsch ME, Shimada K, Skouta R, Viswanathan VS (2014). Regulation of ferroptotic cancer cell death by GPX4. Cell.

[23] Wu G, Fang YZ, Yang S, Lupton JR, Turner ND (2004). Glutathione metabolism and its implications for health. J Nutr.

[24] Zhang H, Forman HJ (2012). Glutathione synthesis and its role in redox signaling. Semin Cell Dev Biol.

[25] Lu SC (1999). Regulation of hepatic glutathione synthesis: current concepts and controversies. FASEB J.

[26] Bannai S, Tateishi N (1986). Role of membrane transport in metabolism and function of glutathione in mammals. J Membr Biol.

[27] Lewerenz J, Hewett SJ, Huang Y, Lambros M, Gout PW, Kalivas PW (2013). The cystine/glutamate antiporter system x(c)(-) in health and disease: from molecular mechanisms to novel therapeutic opportunities. Antioxid Redox Signal.

[28] Fan Z, Wirth AK, Chen D, Wruck CJ, Rauh M, Buchfelder M (2017). Nrf2-Keap1 pathway promotes cell proliferation and diminishes ferroptosis. Oncogenesis..

[29] Durante W. The emerging role of l-glutamine in cardiovascular health and disease. Nutrients. 2019;11:2092. 10.3390/nu11092092 PMC6769761 31487814

[30] Lv H, Zhen C, Liu J, Yang P, Hu L, Shang P (2019). Unraveling the potential role of glutathione in multiple forms of cell death in cancer therapy. Oxid Med Cell Longev.

[31] Lushchak VI (2012). Glutathione homeostasis and functions: potential targets for medical interventions. J Amino Acids.

[32] Gaucher C, Boudier A, Bonetti J, Clarot I, Leroy P, Parent M. Glutathione: antioxidant properties dedicated to nanotechnologies. Antioxidants. 2018;7:62. 10.3390/antiox7050062 PMC5981248 29702624

[33] Krause MS, Oliveira LP, Silveira EMS, Vianna DR, Rossato JS, Almeida BS (2007). MRP1/GS-X pump ATPase expression: is this the explanation for the cytoprotection of the heart against oxidative stress-induced redox imbalance in comparison to skeletal muscle cells?. Cell Biochem Funct.

[34] Jungsuwadee P, Cole MP, Sultana R, Joshi G, Tangpong J, Butterfield DA (2006). Increase in Mrp1 expression and 4-hydroxy-2-nonenal adduction in heart tissue of Adriamycin-treated C57BL/6 mice. Mol Cancer Ther.

[35] Lu SC (2013). Glutathione synthesis. Biochim Biophys Acta.

[36] Dong L-H, Li L, Song Y, Duan Z-L, Sun S-G, Lin Y-L (2015). TRAF6-mediated SM22α K21 ubiquitination promotes G6PD activation and NADPH production, contributing to GSH homeostasis and VSMC survival in vitro and in vivo. Circ Res.

[37] Bachhawat AK, Kaur A (2017). Glutathione degradation. Antioxid Redox Signal.

[38] Bashar T, Akhter N (2014). Study on oxidative stress and antioxidant level in patients of acute myocardial infarction before and after regular treatment. Bangladesh Med Res Counc Bull.

[39] Pechán I, Minárová H, Babusíková F, Rendeková V, Mizera S, Schrameková E (1996). [Parameters of oxidative stress in patients with cardiopathies]. Bratisl Lek Listy.

[40] Chan CY, Mong MC, Liu WH, Huang CY, Yin MC (2014). Three pentacyclic triterpenes protect H9c2 cardiomyoblast cells against high-glucose-induced injury. Free Radic Res.

[41] Liu X, Qi K, Gong Y, Long X, Zhu S, Lu F (2021). Ferulic acid alleviates myocardial ischemia reperfusion injury via upregulating AMPKα2 expression-mediated ferroptosis depression. J Cardiovasc Pharmacol.

[42] Li F, Lang F, Wang Y, Zhai C, Zhang C, Zhang L (2018). Cyanidin ameliorates endotoxin-induced myocardial toxicity by modulating inflammation and oxidative stress through mitochondria and other factors. Food Chem Toxicol.

[43] Li L, Pan Q, Han W, Liu Z, Li L, Hu X (2007). Schisandrin B prevents doxorubicin-induced cardiotoxicity via enhancing glutathione redox cycling. Clin Cancer Res.

[44] Zhao J, Ouyang Y, Wang H, Lai H, Hu S, Tang L (2022). An energy metabolism study on the efficacy of naoxintong capsules against myocardial infarction in a rat model. Oxid Med Cell Longev.

[45] Liao H-H, Zhu J-X, Feng H, Ni J, Zhang N, Chen S (2017). Myricetin possesses potential protective effects on diabetic cardiomyopathy through inhibiting IB/NFB and enhancing Nrf2/HO-1. Oxid Med Cell Longev.

[46] Sudharsan PT, Mythili Y, Selvakumar E, Varalakshmi P (2005). Cardioprotective effect of pentacyclic triterpene, lupeol and its ester on cyclophosphamide-induced oxidative stress. Hum Exp Toxicol.

[47] Shi L, Fu W, Xu H, Li S, Yang X, Yang W (2022). Ginsenoside Rc attenuates myocardial ischaemic injury through antioxidative and anti-inflammatory effects. Pharm Biol.

[48] Wang X, Chen XX, Zhou WQ, Men HB, Bao T, Sun YK (2022). Ferroptosis is essential for diabetic cardiomyopathy and is prevented by sulforaphane via AMPK/NRF2 pathways. Acta Pharm Sin B..

[49] Ning D, Yang X, Wang T, Jiang Q, Yu J, Wang D (2021). Atorvastatin treatment ameliorates cardiac function and remodeling induced by isoproterenol attack through mitigation of ferroptosis. Biochem Biophys Res Commun.

[50] Kumar P, Osahon OW, Sekhar RV. GlyNAC (Glycine and N-Acetylcysteine) Supplementation in mice increases length of life by correcting glutathione deficiency, oxidative stress, mitochondrial dysfunction, abnormalities in mitophagy and nutrient sensing, and genomic damage. Nutrients. 2022;14:1114. 10.3390/nu14051114 PMC8912885 35268089

[51] Ondrejickova O, Ziegelhoeffer A, Gabauer I, Sotnikova R, Styk J, Gibala P (1993). Evaluation of ischemia-reperfusion injury by malondialdehyde, glutathione and gamma-glutamyl transpeptidase: lack of specific local effects in diverse parts of the dog heart following acute coronary occlusion. Cardioscience..

[52] Zheng M-Q, Tang K, Zimmerman MC, Liu L, Xie B, Rozanski GJ (2009). Role of gamma-glutamyl transpeptidase in redox regulation of K+ channel remodeling in postmyocardial infarction rat hearts. Am J Physiol Cell Physiol.

[53] Kitakata H, Endo J, Matsushima H, Yamamoto S, Ikura H, Hirai A (2021). MITOL/MARCH5 determines the susceptibility of cardiomyocytes to doxorubicin-induced ferroptosis by regulating GSH homeostasis. J Mol Cell Cardiol.

[54] Dorn GW, Vega RB, Kelly DP (2015). Mitochondrial biogenesis and dynamics in the developing and diseased heart. Genes Dev.

[55] Peoples JN, Saraf A, Ghazal N, Pham TT, Kwong JQ. Mitochondrial dysfunction and oxidative stress in heart disease. Exp Mol Med. 2019;51:1–13. 10.1038/s12276-019-0355-7 PMC6923355 31857574

[56] Cadenas E, Davies KJ (2000). Mitochondrial free radical generation, oxidative stress, and aging. Free Radic Biol Med.

[57] Ribas V, García-Ruiz C, Fernández-Checa JC (2014). Glutathione and mitochondria. Front Pharmacol.

[58] Marí M, Morales A, Colell A, García-Ruiz C, Fernández-Checa JC (2009). Mitochondrial glutathione, a key survival antioxidant. Antioxid Redox Signal.

[59] Lillo-Moya J, Rojas-Solé C, Muñoz-Salamanca D, Panieri E, Saso L, Rodrigo R. Targeting ferroptosis against ischemia/reperfusion cardiac injury. Antioxidants. 2021;10:667. 10.3390/antiox10050667 PMC8145541 33922912

[60] Jang S, Chapa-Dubocq XR, Tyurina YY, St Croix CM, Kapralov AA, Tyurin VA (2021). Elucidating the contribution of mitochondrial glutathione to ferroptosis in cardiomyocytes. Redox Biol.

[61] Ta N, Qu C, Wu H, Zhang D, Sun T, Li Y (2022). Mitochondrial outer membrane protein FUNDC2 promotes ferroptosis and contributes to doxorubicin-induced cardiomyopathy. Proc Natl Acad Sci USA.

[62] Wang Y, Yen FS, Zhu XG, Timson RC, Weber R, Xing C (2021). SLC25A39 is necessary for mitochondrial glutathione import in mammalian cells. Nature..

[63] Venter H, Genade S, Mouton R, Huisamen B, Harper IS, Lochner A (1991). Myocardial membrane cholesterol: effects of ischaemia. J Mol Cell Cardiol.

[64] Brigelius-Flohe R, Flohe L (2020). Regulatory phenomena in the glutathione peroxidase superfamily. Antioxid Redox Signal.

[65] Brigelius-Flohe R, Maiorino M (2013). Glutathione peroxidases. Biochim Biophys Acta.

[66] Chen X, Li J, Kang R, Klionsky DJ, Tang D (2021). Ferroptosis: machinery and regulation. Autophagy..

[67] Zhu M, Wang H, Chen J, Zhu H (2021). Sinomenine improve diabetic nephropathy by inhibiting fibrosis and regulating the JAK2/STAT3/SOCS1 pathway in streptozotocin-induced diabetic rats. Life Sci.

[68] Labunskyy VM, Hatfield DL, Gladyshev VN (2014). Selenoproteins: molecular pathways and physiological roles. Physiol Rev.

[69] Fang X, Ardehali H, Min J, Wang F. The molecular and metabolic landscape of iron and ferroptosis in cardiovascular disease. Nat Rev Cardiol. 2022;20:7–23. 10.1038/s41569-022-00735-4 PMC9252571 35788564

[70] Koleini N, Shapiro JS, Geier J, Ardehali H. Ironing out mechanisms of iron homeostasis and disorders of iron deficiency. J Clin Invest. 2021;131:e148671. 10.1172/JCI148671 PMC8159681 34060484

[71] Luck AN, Mason AB. Transferrin-mediated cellular iron delivery. Curr Top Membr. 2012;69:3–35. 10.1016/B978-0-12-394390-3.00001-X PMC4479283 23046645

[72] Hong M, Rong J, Tao X, Xu Y (2022). The emerging role of ferroptosis in cardiovascular diseases. Front Pharm.

[73] Xie Y, Hou W, Song X, Yu Y, Huang J, Sun X (2016). Ferroptosis: process and function. Cell Death Differ.

[74] Vela D (2018). Balance of cardiac and systemic hepcidin and its role in heart physiology and pathology. Lab Invest.

[75] Feng H, Stockwell BR (2018). Unsolved mysteries: how does lipid peroxidation cause ferroptosis?. PLoS Biol.

[76] Fang X, Cai Z, Wang H, Han D, Cheng Q, Zhang P (2020). Loss of cardiac ferritin H facilitates cardiomyopathy via Slc7a11-mediated ferroptosis. Circ Res.

[77] Tang L-J, Zhou Y-J, Xiong X-M, Li N-S, Zhang J-J, Luo X-J (2021). Ubiquitin-specific protease 7 promotes ferroptosis via activation of the p53/TfR1 pathway in the rat hearts after ischemia/reperfusion. Free Radic Biol Med.

[78] Feng H, Schorpp K, Jin J, Yozwiak CE, Hoffstrom BG, Decker AM, et al. Transferrin receptor is a specific ferroptosis marker. Cell Rep. 2020;30:3411–23.e7. 10.1016/j.celrep.2020.02.049 PMC7172030 32160546

[79] Fan Z, Cai L, Wang S, Wang J, Chen B (2021). Baicalin prevents myocardial ischemia/reperfusion injury through inhibiting ACSL4 mediated ferroptosis. Front Pharmacol.

[80] Kuhn H, Banthiya S, van Leyen K (2015). Mammalian lipoxygenases and their biological relevance. Biochim Biophys Acta.

[81] Wenzel SE, Tyurina YY, Zhao J, St Croix CM, Dar HH, Mao G, et al. PEBP1 Wardens Ferroptosis by Enabling Lipoxygenase Generation of Lipid Death Signals. Cell. 2017;171:628–41.e26. 10.1016/j.cell.2017.09.044 PMC5683852 29053969

[82] Bersuker K, Hendricks JM, Li Z, Magtanong L, Ford B, Tang PH (2019). The CoQ oxidoreductase FSP1 acts parallel to GPX4 to inhibit ferroptosis. Nature..

[83] Doll S, Freitas FP, Shah R, Aldrovandi M, da Silva MC, Ingold I (2019). FSP1 is a glutathione-independent ferroptosis suppressor. Nature..

[84] Miriyala S, Thippakorn C, Chaiswing L, Xu Y, Noel T, Tovmasyan A (2016). Novel role of 4-hydroxy-2-nonenal in AIFm2-mediated mitochondrial stress signaling. Free Radic Biol Med.

[85] Shin D, Lee J, You JH, Kim D, Roh J-L (2020). Dihydrolipoamide dehydrogenase regulates cystine deprivation-induced ferroptosis in head and neck cancer. Redox Biol.

[86] Gao M, Monian P, Quadri N, Ramasamy R, Jiang X (2015). Glutaminolysis and transferrin regulate ferroptosis. Mol Cell.

[87] Park TJ, Park JH, Lee GS, Lee JY, Shin JH, Kim MW (2019). Quantitative proteomic analyses reveal that GPX4 downregulation during myocardial infarction contributes to ferroptosis in cardiomyocytes. Cell Death Dis.

[88] Tang LJ, Luo XJ, Tu H, Chen H, Xiong XM, Li NS (2021). Ferroptosis occurs in phase of reperfusion but not ischemia in rat heart following ischemia or ischemia/reperfusion. Naunyn Schmiedebergs Arch Pharm.

[89] Zhang J, Liu D, Zhang M, Zhang Y (2019). Programmed necrosis in cardiomyocytes: mitochondria, death receptors and beyond. Br J Pharm.

[90] Lu H, Xiao H, Dai M, Xue Y, Zhao R (2022). Britanin relieves ferroptosis-mediated myocardial ischaemia/reperfusion damage by upregulating GPX4 through activation of AMPK/GSK3β/Nrf2 signalling. Pharm Biol.

[91] Fang X, Wang H, Han D, Xie E, Yang X, Wei J (2019). Ferroptosis as a target for protection against cardiomyopathy. Proc Natl Acad Sci USA.

[92] Li D, Liu X, Pi W, Zhang Y, Yu L, Xu C (2021). Fisetin attenuates doxorubicin-induced cardiomyopathy and by inhibiting ferroptosis through SIRT1/Nrf2 signaling pathway activation. Front Pharmacol.

[93] Kong C, Ni X, Wang Y, Zhang A, Zhang Y, Lin F (2022). ICA69 aggravates ferroptosis causing septic cardiac dysfunction via STING trafficking. Cell Death Disco.

[94] Wang C, Yuan W, Hu A, Lin J, Xia Z, Yang CF (2020). Dexmedetomidine alleviated sepsis‑induced myocardial ferroptosis and septic heart injury. Mol Med Rep.

[95] Ni T, Huang X, Pan S, Lu Z (2021). Inhibition of the long non-coding RNA ZFAS1 attenuates ferroptosis by sponging miR-150-5p and activates CCND2 against diabetic cardiomyopathy. J Cell Mol Med.

[96] Zhang Z, Tang J, Song J, Xie M, Liu Y, Dong Z (2022). Elabela alleviates ferroptosis, myocardial remodeling, fibrosis and heart dysfunction in hypertensive mice by modulating the IL-6/STAT3/GPX4 signaling. Free Radic Biol Med.

[97] Zhang X, Zheng C, Gao Z, Chen H, Li K, Wang L (2022). SLC7A11/xCT prevents cardiac hypertrophy by inhibiting ferroptosis. Cardiovasc Drugs Ther.

[98] Sinha R, Sinha I, Calcagnotto A, Trushin N, Haley JS, Schell TD (2018). Oral supplementation with liposomal glutathione elevates body stores of glutathione and markers of immune function. Eur J Clin Nutr.

[99] Pizzorno J (2014). Glutathione!. Integr Med.

[100] Basha RH, Priscilla DH (2013). An in vivo and in vitro study on the protective effects of N-acetylcysteine on mitochondrial dysfunction in isoproterenol treated myocardial infarcted rats. Exp Toxicol Pathol.

[101] Rushworth GF, Megson IL (2014). Existing and potential therapeutic uses for N-acetylcysteine: the need for conversion to intracellular glutathione for antioxidant benefits. Pharm Ther.

[102] Arstall MA, Yang J, Stafford I, Betts WH, Horowitz JD (1995). N-acetylcysteine in combination with nitroglycerin and streptokinase for the treatment of evolving acute myocardial infarction. Saf Biochem Eff Circ.

[103] Pasupathy S, Tavella R, Grover S, Raman B, Procter NEK, Du YT (2017). Early use of n-acetylcysteine with nitrate therapy in patients undergoing primary percutaneous coronary intervention for ST-segment-elevation myocardial infarction reduces myocardial infarct size (the NACIAM Trial [N-acetylcysteine in Acute Myocardial Infarction]). Circulation..

[104] Falkowski M, Maciejczyk M, Koprowicz T, Mikoluc B, Milewska A, Zalewska A, et al. Whey protein concentrate WPC-80 improves antioxidant defense systems in the salivary glands of 14-month wistar rats. Nutrients. 2018;10:782. 10.3390/nu10060782 PMC6024865 29914217

[105] Bartfay WJ, Davis MT, Medves JM, Lugowski S (2003). Milk whey protein decreases oxygen free radical production in a murine model of chronic iron-overload cardiomyopathy. Can J Cardiol.

[106] Jakubowski H (2019). Homocysteine modification in protein structure/function and human disease. Physiol Rev.

[107] Singh AP, Singh M, Balakumar P (2008). Effect of mast cell stabilizers in hyperhomocysteinemia-induced cardiac hypertrophy in rats. J Cardiovasc Pharm.

[108] Zhang Y, Lv SJ, Yan H, Wang L, Liang GP, Wan QX (2013). Effects of glycine supplementation on myocardial damage and cardiac function after severe burn. Burns..

[109] Cieslik KA, Sekhar RV, Granillo A, Reddy A, Medrano G, Heredia CP (2018). Improved cardiovascular function in old mice after N-Acetyl cysteine and glycine supplemented diet: inflammation and mitochondrial factors. J Gerontol A Biol Sci Med Sci.

[110] Kumar P, Liu C, Hsu JW, Chacko S, Minard C, Jahoor F (2021). Glycine and N-acetylcysteine (GlyNAC) supplementation in older adults improves glutathione deficiency, oxidative stress, mitochondrial dysfunction, inflammation, insulin resistance, endothelial dysfunction, genotoxicity, muscle strength, and cognition: results of a pilot clinical trial. Clin Transl Med.

[111] H S Kumar S, Anandan R (2007). Biochemical studies on the cardioprotective effect of glutamine on tissue antioxidant defense system in isoprenaline-induced myocardial infarction in rats. J Clin Biochem Nutr.

[112] Todorova V, Vanderpool D, Blossom S, Nwokedi E, Hennings L, Mrak R (2009). Oral glutamine protects against cyclophosphamide-induced cardiotoxicity in experimental rats through increase of cardiac glutathione. Nutrition.

[113] Engel JM, Muhling J, Kwapisz M, Heidt M (2009). Glutamine administration in patients undergoing cardiac surgery and the influence on blood glutathione levels. Acta Anaesthesiol Scand.

[114] Zhu H, Wang X, Meng X, Kong Y, Li Y, Yang C (2022). Selenium Supplementation Improved Cardiac Functions by Suppressing DNMT2-Mediated GPX1 Promoter DNA Methylation in AGE-Induced Heart Failure. Oxid Med Cell Longev.

[115] Tanguy S, Morel S, Berthonneche C, Toufektsian M-C, de Lorgeril M, Ducros V (2004). Preischemic selenium status as a major determinant of myocardial infarct size in vivo in rats. Antioxid Redox Signal.

[116] Kuria A, Tian H, Li M, Wang Y, Aaseth JO, Zang J (2021). Selenium status in the body and cardiovascular disease: a systematic review and meta-analysis. Crit Rev Food Sci Nutr.

[117] Cardoso BR, Cominetti C, Seale LA (2021). Editorial: selenium, human health and chronic disease. Front Nutr.

[118] Jenkins DJA, Kitts D, Giovannucci EL, Sahye-Pudaruth S, Paquette M, Blanco Mejia S (2020). Selenium, antioxidants, cardiovascular disease, and all-cause mortality: a systematic review and meta-analysis of randomized controlled trials. Am J Clin Nutr.

[119] Al Numair KS, Chandramohan G, Alsaif MA, Baskar AA (2012). Protective effect of morin on cardiac mitochondrial function during isoproterenol-induced myocardial infarction in male Wistar rats. Redox Rep.

[120] Liu X, Yu Z, Huang X, Gao Y, Wang X, Gu J (2016). Peroxisome proliferator-activated receptor γ (PPARγ) mediates the protective effect of quercetin against myocardial ischemia-reperfusion injury via suppressing the NF-κB pathway. Am J Transl Res.

[121] Saleh Ahmed AS. Potential protective effect of catechin on doxorubicin-induced cardiotoxicity in adult male albino rats. Toxicol Mech Methods. 2022;32:97–105. 10.1080/15376516.2021.1972375 34427160

[122] Panche AN, Diwan AD, Chandra SR (2016). Flavonoids: an overview. J Nutr Sci.

[123] Liu H, Guo X, Chu Y, Lu S (2014). Heart protective effects and mechanism of quercetin preconditioning on anti-myocardial ischemia reperfusion (IR) injuries in rats. Gene..

[124] El-Sayed SS, Shahin RM, Fahmy A, Elshazly SM (2021). Quercetin ameliorated remote myocardial injury induced by renal ischemia/reperfusion in rats: Role of Rho-kinase and hydrogen sulfide. Life Sci.

[125] Roslan J, Giribabu N, Karim K, Salleh N (2017). Quercetin ameliorates oxidative stress, inflammation and apoptosis in the heart of streptozotocin-nicotinamide-induced adult male diabetic rats. Biomed Pharmacother.

[126] Dehghani F, Sezavar Seyedi Jandaghi SH, Janani L, Sarebanhassanabadi M, Emamat H, Vafa M (2021). Effects of quercetin supplementation on inflammatory factors and quality of life in post-myocardial infarction patients: a double blind, placebo-controlled, randomized clinical trial. Phytother Res.

[127] Chekalina NI, Shut SV, Trybrat TA, Burmak YH, Petrov YY, Manusha YI (2017). Effect of quercetin on parameters of central hemodynamics and myocardial ischemia in patients with stable coronary heart disease. Wiad Lek.

[128] Ranjan A, Ramachandran S, Gupta N, Kaushik I, Wright S, Srivastava S, et al. Role of phytochemicals in cancer prevention. Int J Mol Sci. 2019;20:4981. 10.3390/ijms20204981 PMC6834187 31600949

[129] Prince PSM. A biochemical, electrocardiographic, electrophoretic, histopathological and in vitro study on the protective effects of (-)epicatechin in isoproterenol-induced myocardial infarcted rats. Eur J Pharmacol. 2011;671:95–101. 10.1016/j.ejphar.2011.09.036 21958876

[130] Devika PT, Stanely Mainzen Prince P (2008). Protective effect of (-)-epigallocatechin-gallate (EGCG) on lipid peroxide metabolism in isoproterenol induced myocardial infarction in male Wistar rats: a histopathological study. Biomed Pharmacother.

[131] Hu Q-P, Yan H-X, Peng F, Feng W, Chen F-F, Huang X-Y (2021). Genistein protects epilepsy-induced brain injury through regulating the JAK2/STAT3 and Keap1/Nrf2 signaling pathways in the developing rats. Eur J Pharm.

[132] Bai Z, Wang Z (2019). Genistein protects against doxorubicin-induced cardiotoxicity through Nrf-2/HO-1 signaling in mice model. Environ Toxicol.

[133] Jia Q, Wang Y, Liu X, Ma S, Yang R. Effects of genistein on Nrf2/HO-1 pathway in myocardial tissues of diabetic rats. Zhong Nan Da Xue Xue Bao Yi Xue Ban. 2019;44:850–6. 10.11817/j.issn.1672-7347.2019.08.180429 31570670

[134] Jia Q, Yang R, Liu X-F, Ma S-F (2018). Protective effects of genistein on myocardial injury in diabetic rats. Sichuan Da Xue Xue Bao Yi Xue Ban.

[135] Zhai X, Lin M, Zhang F, Hu Y, Xu X, Li Y (2013). Dietary flavonoid genistein induces Nrf2 and phase II detoxification gene expression via ERKs and PKC pathways and protects against oxidative stress in Caco-2 cells. Mol Nutr Food Res.

[136] Luo M, Zheng L-W, Wang Y-S, Huang J-C, Yang Z-Q, Yue Z-P (2021). Genistein exhibits therapeutic potential for PCOS mice via the ER-Nrf2-Foxo1-ROS pathway. Food Funct.

[137] Niu L-G, Sun N, Liu K-L, Su Q, Qi J, Fu L-Y (2022). Genistein alleviates oxidative stress and inflammation in the hypothalamic paraventricular nucleus by activating the Sirt1/Nrf2 pathway in high salt-induced hypertension. Cardiovasc Toxicol.

[138] De Gregorio C, Marini H, Alibrandi A, Di Benedetto A, Bitto A, Adamo EB, et al. Genistein supplementation and cardiac function in postmenopausal women with metabolic syndrome: results from a pilot strain-echo study. Nutrients. 2017;9:584. 10.3390/nu9060584 PMC5490563 28590452

[139] Li Y, Zhang H (2017). Soybean isoflavones ameliorate ischemic cardiomyopathy by activating Nrf2-mediated antioxidant responses. Food Funct.

[140] Proshkina E, Plyusnin S, Babak T, Lashmanova E, Maganova F, Koval L, et al. Terpenoids as potential geroprotectors. Antioxidants. 2020;9:529. 10.3390/antiox9060529 PMC7346221 32560451

[141] Milani A, Basirnejad M, Shahbazi S, Bolhassani A (2017). Carotenoids: biochemistry, pharmacology and treatment. Br J Pharm.

[142] Bohn T, Desmarchelier C, El SN, Keijer J, van Schothorst E, Ruhl R (2019). beta-Carotene in the human body: metabolic bioactivation pathways - from digestion to tissue distribution and excretion. Proc Nutr Soc.

[143] Maritim A, Dene BA, Sanders RA, Watkins JB (2002). Effects of beta-carotene on oxidative stress in normal and diabetic rats. J Biochem Mol Toxicol.

[144] Zhao G, Zhang X, Wang H, Chen Z (2020). Beta carotene protects H9c2 cardiomyocytes from advanced glycation end product-induced endoplasmic reticulum stress, apoptosis, and autophagy via the PI3K/Akt/mTOR signaling pathway. Ann Transl Med.

[145] Csepanyi E, Czompa A, Haines D, Lekli I, Bakondi E, Balla G (2015). Cardiovascular effects of low versus high-dose beta-carotene in a rat model. Pharm Res.

[146] Panczenko-Kresowska B, Ziemlański S, Rudnicki S, Wojtulewicz L, Przepiórka M (1998). The influence of vitamin C and e or beta-carotene on peroxidative processes in persons with myocardial ischemia. Pol Merkur Lekarski.

[147] Schwingshackl L, Boeing H, Stelmach-Mardas M, Gottschald M, Dietrich S, Hoffmann G (2017). Dietary supplements and risk of cause-specific death, cardiovascular disease, and cancer: a systematic review and meta-analysis of primary prevention trials. Adv Nutr (Bethesda, Md).

[148] Qiu Z, Chen X, Geng T, Wan Z, Lu Q, Li L (2022). Associations of Serum Carotenoids With Risk of Cardiovascular Mortality Among Individuals With Type 2 Diabetes: Results From NHANES. Diabetes Care.

[149] Neyestani TR, Shariatzadeh N, Gharavi A, Kalayi A, Khalaji N (2007). Physiological dose of lycopene suppressed oxidative stress and enhanced serum levels of immunoglobulin M in patients with Type 2 diabetes mellitus: a possible role in the prevention of long-term complications. J Endocrinol Invest.

[150] Zheng S, Deng Z, Chen F, Zheng L, Pan Y, Xing Q (2020). Synergistic antioxidant effects of petunidin and lycopene in H9c2 cells submitted to hydrogen peroxide: Role of Akt/Nrf2 pathway. J Food Sci.

[151] Abdel-Daim MM, Eltaysh R, Hassan A, Mousa SA. Lycopene attenuates tulathromycin and diclofenac sodium-induced cardiotoxicity in mice. Int J Mol Sci. 2018;19:344. 10.3390/ijms19020344 PMC5855566 29364179

[152] Yilmaz S, Atessahin A, Sahna E, Karahan I, Ozer S (2006). Protective effect of lycopene on adriamycin-induced cardiotoxicity and nephrotoxicity. Toxicology..

[153] Shao H, Fang C, Huang Y, Ye Y, Tong R (2022). Sodium tanshinone IIA sulfonate injection as adjunctive therapy for the treatment of heart failure: a systematic review and meta-analysis. Phytomedicine.

[154] Guo Z, Yan M, Chen L, Fang P, Li Z, Wan Z (2018). Nrf2-dependent antioxidant response mediated the protective effect of tanshinone IIA on doxorubicin-induced cardiotoxicity. Exp Ther Med.

[155] Hu H, Zhai C, Qian G, Gu A, Liu J, Ying F (2015). Protective effects of tanshinone IIA on myocardial ischemia reperfusion injury by reducing oxidative stress, HMGB1 expression, and inflammatory reaction. Pharm Biol.

[156] Mao S, Wang L, Zhao X, Guo L, Lin Q, Wang X (2021). Efficacy of sodium tanshinone IIA sulfonate in patients with non-ST elevation acute coronary syndrome undergoing percutaneous coronary intervention: results from a multicentre, controlled, randomized trial. Cardiovasc Drugs Ther.

[157] Neelam, Khatkar A, Sharma KK (2020). Phenylpropanoids and its derivatives: biological activities and its role in food, pharmaceutical and cosmetic industries. Crit Rev Food Sci Nutr.

[158] Anupama N, Preetha Rani MR, Shyni GL, Raghu KG (2018). Glucotoxicity results in apoptosis in H9c2 cells via alteration in redox homeostasis linked mitochondrial dynamics and polyol pathway and possible reversal with cinnamic acid. Toxicol Vitr.

[159] Kumaran KS, Prince PSM (2010). Protective effect of caffeic acid on cardiac markers and lipid peroxide metabolism in cardiotoxic rats: an in vivo and in vitro study. Metabolism..

[160] Akila P, Vennila L (2016). Chlorogenic acid a dietary polyphenol attenuates isoproterenol induced myocardial oxidative stress in rat myocardium: an in vivo study. Biomed Pharmacother.

[161] Li H, Xie Y-H, Yang Q, Wang S-W, Zhang B-L, Wang J-B (2012). Cardioprotective effect of paeonol and danshensu combination on isoproterenol-induced myocardial injury in rats. PLoS One.

[162] Sammeturi M, Shaik AH, Bongu SBR, Cheemanapalli S, Mohammad A, Kodidhela LD (2019). Protective effects of syringic acid, resveratrol and their combination against isoprenaline administered cardiotoxicity in wistar rats. Saudi J Biol Sci.

[163] Priscilla DH, Prince PSM (2009). Cardioprotective effect of gallic acid on cardiac troponin-T, cardiac marker enzymes, lipid peroxidation products and antioxidants in experimentally induced myocardial infarction in Wistar rats. Chem Biol Interact.

[164] Chao P-C, Hsu C-C, Yin M-C (2009). Anti-inflammatory and anti-coagulatory activities of caffeic acid and ellagic acid in cardiac tissue of diabetic mice. Nutr Metab.

[165] Yeh C-T, Ching L-C, Yen G-C (2009). Inducing gene expression of cardiac antioxidant enzymes by dietary phenolic acids in rats. J Nutr Biochem.

[166] Li G, Huang X (2021). Influence of sodium ferulate on miR-133a and left ventricle remodeling in rats with myocardial infarction. Hum Exp Toxicol.

[167] Wang B-H, Ou-Yang J-P (2005). Pharmacological actions of sodium ferulate in cardiovascular system. Cardiovasc Drug Rev.

[168] Socała K, Szopa A, Serefko A, Poleszak E, Wlaź P. Neuroprotective effects of coffee bioactive compounds: a review. Int J Mol Sci. 2020;22:107. 10.3390/ijms22010107 PMC7795778 33374338

[169] Martínez-López S, Sarriá B, Mateos R, Bravo-Clemente L (2019). Moderate consumption of a soluble green/roasted coffee rich in caffeoylquinic acids reduces cardiovascular risk markers: results from a randomized, cross-over, controlled trial in healthy and hypercholesterolemic subjects. Eur J Nutr.

[170] Sato Y, Itagaki S, Kurokawa T, Ogura J, Kobayashi M, Hirano T (2011). In vitro and in vivo antioxidant properties of chlorogenic acid and caffeic acid. Int J Pharm.

[171] Yüce A, Ateşşahin A, Ceribaşi AO, Aksakal M (2007). Ellagic acid prevents cisplatin-induced oxidative stress in liver and heart tissue of rats. Basic Clin Pharmacol Toxicol.

[172] Ghadimi M, Foroughi F, Hashemipour S, Rashidi Nooshabadi M, Ahmadi MH, Ahadi Nezhad B (2021). Randomized double-blind clinical trial examining the Ellagic acid effects on glycemic status, insulin resistance, antioxidant, and inflammatory factors in patients with type 2 diabetes. Phytother Res.

[173] Zhang M, Cui S, Mao B, Zhang Q, Zhao J, Zhang H, et al. Ellagic acid and intestinal microflora metabolite urolithin A: a review on its sources, metabolic distribution, health benefits, and biotransformation. Critical Rev Food Sci Nutrition. 2022;10:1–23. 10.1080/10408398.2022.2036693 35142569

[174] Albasher G, Alkahtani S, Al-Harbi LN (2022). Urolithin A prevents streptozotocin-induced diabetic cardiomyopathy in rats by activating SIRT1. Saudi J Biol Sci.

[175] Ayer A, Macdonald P, Stocker R. CoQ_10_ function and role in heart failure and ischemic heart disease. Annu Rev Nutr. 2015;35:175–213. 10.1146/annurev-nutr-071714-034258 25974695

[176] Rabanal-Ruiz Y, Llanos-González E, Alcain FJ. The use of coenzyme q10 in cardiovascular diseases. Antioxidants. 2021;10:755. 10.3390/antiox10050755 PMC8151454 34068578

[177] Ghule AE, Kulkarni CP, Bodhankar SL, Pandit VA (2009). Effect of pretreatment with coenzyme Q10 on isoproterenol-induced cardiotoxicity and cardiac hypertrophy in rats. Curr Ther Res Clin Exp.

[178] Lee B-J, Tseng Y-F, Yen C-H, Lin P-T (2013). Effects of coenzyme Q10 supplementation (300 mg/day) on antioxidation and anti-inflammation in coronary artery disease patients during statins therapy: a randomized, placebo-controlled trial. Nutr J.

[179] Di Pierro D, Ciaccio C, Sbardella D, Tundo GR, Bernardini R, Curatolo P (2020). Effects of oral administration of common antioxidant supplements on the energy metabolism of red blood cells. Attenuation of oxidative stress-induced changes in Rett syndrome erythrocytes by CoQ10. Mol Cell Biochem.

[180] Danaei GH, Memar B, Ataee R, Karami M (2019). Protective effect of thymoquinone, the main component of, against diazinon cardio-toxicity in rats. Drug Chem Toxicol.

[181] Shoaei-Hagh P, Kamelan Kafi F, Najafi S, Zamanzadeh M, Heidari Bakavoli A, Ramezani J (2021). A randomized, double-blind, placebo-controlled, clinical trial to evaluate the benefits of Nigella sativa seeds oil in reducing cardiovascular risks in hypertensive patients. Phytother Res.

[182] Nazari Soltan Ahmad S, Sanajou D, Kalantary-Charvadeh A, Hosseini V, Roshangar L, Khojastehfard M (2020). beta-LAPachone ameliorates doxorubicin-induced cardiotoxicity via regulating autophagy and Nrf2 signalling pathways in mice. Basic Clin Pharm Toxicol.

[183] Birari L, Wagh S, Patil KR, Mahajan UB, Unger B, Belemkar S (2020). Aloin alleviates doxorubicin-induced cardiotoxicity in rats by abrogating oxidative stress and pro-inflammatory cytokines. Cancer Chemother Pharm.

[184] Mishra P, Paital B, Jena S, Swain SS, Kumar S, Yadav MK (2019). Possible activation of NRF2 by Vitamin E/Curcumin against altered thyroid hormone induced oxidative stress via NFĸB/AKT/mTOR/KEAP1 signalling in rat heart. Sci Rep.

[185] Wang R, Zhang JY, Zhang M, Zhai MG, Di SY, Han QH (2018). Curcumin attenuates IR-induced myocardial injury by activating SIRT3. Eur Rev Med Pharm Sci.

[186] Chen R, Peng X, Du W, Wu Y, Huang B, Xue L (2015). Curcumin attenuates cardiomyocyte hypertrophy induced by high glucose and insulin via the PPARγ/Akt/NO signaling pathway. Diabetes Res Clin Pr.

[187] Mohammed HS, Hosny EN, Khadrawy YA, Magdy M, Attia YS, Sayed OA (2020). Protective effect of curcumin nanoparticles against cardiotoxicity induced by doxorubicin in rat. Biochim Biophys Acta Mol Basis Dis.

[188] Sarawi WS, Alhusaini AM, Fadda LM, Alomar HA, Albaker AB, Aljrboa AS, et al. Nano-curcumin prevents cardiac injury, oxidative stress and inflammation, and modulates TLR4/NF-κB and MAPK signaling in copper sulfate-intoxicated rats. Antioxidants. 2021;10:1414. 10.3390/antiox10091414 PMC8469340 34573046

[189] Shafabakhsh R, Mobini M, Raygan F, Aghadavod E, Ostadmohammadi V, Amirani E (2020). Curcumin administration and the effects on psychological status and markers of inflammation and oxidative damage in patients with type 2 diabetes and coronary heart disease. Clin Nutr ESPEN.

[190] Helli B, Gerami H, Kavianpour M, Heybar H, Hosseini SK, Haghighian HK (2021). Curcumin nanomicelle improves lipid profile, stress oxidative factors and inflammatory markers in patients undergoing coronary elective angioplasty; a randomized clinical trial. Endocr Metab Immune Disord Drug Targets.

[191] Xia N, Daiber A, Forstermann U, Li H (2017). Antioxidant effects of resveratrol in the cardiovascular system. Br J Pharm.

[192] Tatlidede E, Sehirli O, Velioğlu-Oğünc A, Cetinel S, Yeğen BC, Yarat A (2009). Resveratrol treatment protects against doxorubicin-induced cardiotoxicity by alleviating oxidative damage. Free Radic Res.

[193] Wang X, Simayi A, Fu J, Zhao X, Xu G. Resveratrol mediates the miR-149/HMGB1 axis and regulates the ferroptosis pathway to protect myocardium in endotoxemia mice. Am J Physiol Endocrinol Metab. 2022;323:E21–E32. 10.1152/ajpendo.00227.2021 35532075

[194] Ibrahim KA, Abdelgaid HA, Eleyan M, Mohamed RA, Gamil NM (2022). Resveratrol alleviates cardiac apoptosis following exposure to fenitrothion by modulating the sirtuin1/c-Jun N-terminal kinases/p53 pathway through pro-oxidant and inflammatory response improvements: In vivo and in silico studies. Life Sci.

[195] Liu J, Zhang M, Qin C, Wang Z, Chen J, Wang R (2022). Resveratrol attenuate myocardial injury by inhibiting ferroptosis inducing KAT5/GPX4 in myocardial Infarction. Front Pharmacol.

[196] Xu G, Zhao X, Fu J, Wang X (2019). Resveratrol increase myocardial Nrf2 expression in type 2 diabetic rats and alleviate myocardial ischemia/reperfusion injury (MIRI). Ann Palliat Med.

[197] Guo S, Yao Q, Ke Z, Chen H, Wu J, Liu C (2015). Resveratrol attenuates high glucose-induced oxidative stress and cardiomyocyte apoptosis through AMPK. Mol Cell Endocrinol.

[198] Hoseini A, Namazi G, Farrokhian A, Reiner Ž, Aghadavod E, Bahmani F (2019). The effects of resveratrol on metabolic status in patients with type 2 diabetes mellitus and coronary heart disease. Food Funct.

[199] Chekalina NI (2017). Resveratrol has a positive effect on parameters of central hemodynamics and myocardial ischemia in patients with stable coronary heart disease. Wiad Lek.

[200] Gorrini C, Harris IS, Mak TW (2013). Modulation of oxidative stress as an anticancer strategy. Nat Rev Drug Disco.

[201] Zhu H, Itoh K, Yamamoto M, Zweier JL, Li Y (2005). Role of Nrf2 signaling in regulation of antioxidants and phase 2 enzymes in cardiac fibroblasts: protection against reactive oxygen and nitrogen species-induced cell injury. Febs Lett.

[202] Shanmugam G, Challa AK, Litovsky SH, Devarajan A, Wang D, Jones DP (2019). Enhanced Keap1-Nrf2 signaling protects the myocardium from isoproterenol-induced pathological remodeling in mice. Redox Biol.

[203] Bai Y, Guo J, Reiter RJ, Wei Y, Shi H (2020). Melatonin synthesis enzymes interact with ascorbate peroxidase to protect against oxidative stress in cassava. J Exp Bot.

[204] Vriend J, Reiter RJ (2015). The Keap1-Nrf2-antioxidant response element pathway: a review of its regulation by melatonin and the proteasome. Mol Cell Endocrinol.

[205] Zhi W, Li K, Wang H, Lei M, Guo Y (2020). Melatonin elicits protective effects on OGD/R‑insulted H9c2 cells by activating PGC‑1α/Nrf2 signaling. Int J Mol Med.

[206] Cai J, Yang J, Chen X, Zhang H, Zhu Y, Liu Q (2022). Melatonin ameliorates trimethyltin chloride-induced cardiotoxicity: the role of nuclear xenobiotic metabolism and Keap1-Nrf2/ARE axis-mediated pyroptosis. Biofactors..

[207] Lan H, Su Y, Liu Y, Deng C, Wang J, Chen T (2019). Melatonin protects circulatory death heart from ischemia/reperfusion injury via the JAK2/STAT3 signalling pathway. Life Sci.

[208] Haghjooy Javanmard S, Ziaei A, Ziaei S, Ziaei E, Mirmohammad-Sadeghi M (2013). The effect of preoperative melatonin on nuclear erythroid 2-related factor 2 activation in patients undergoing coronary artery bypass grafting surgery. Oxid Med Cell Longev.

[209] Raygan F, Ostadmohammadi V, Bahmani F, Reiter RJ, Asemi Z (2019). Melatonin administration lowers biomarkers of oxidative stress and cardio-metabolic risk in type 2 diabetic patients with coronary heart disease: a randomized, double-blind, placebo-controlled trial. Clin Nutr.

[210] Zhang H, Liu M, Zhang Y, Li X (2019). Trimetazidine attenuates exhaustive exercise-induced myocardial injury in rats via regulation of the Nrf2/NF-κB signaling pathway. Front Pharmacol.

[211] Wu S, Chang G, Gao L, Jiang D, Wang L, Li G (2018). Trimetazidine protects against myocardial ischemia/reperfusion injury by inhibiting excessive autophagy. J Mol Med.

[212] Eid BG, El-Shitany NAE-A, Neamatallah T (2021). Trimetazidine improved adriamycin-induced cardiomyopathy by downregulating TNF-α, BAX, and VEGF immunoexpression via an antioxidant mechanism. Environ Toxicol.

[213] Ramezani-Aliakbari F, Badavi M, Dianat M, Mard SA, Ahangarpour A (2019). The effects of trimetazidine on QT-interval prolongation and cardiac hypertrophy in diabetic rats. Arq Bras Cardiol.

[214] Zhang L, Wu P, Zhang L, SreeHarsha N, Mishra A, Su X (2020). Ameliorative effect of rosiglitazone, a peroxisome proliferator gamma agonist on adriamycin-induced cardio toxicity via suppressing oxidative stress and apoptosis. IUBMB Life.

[215] Gumieniczek A (2005). Modification of oxidative stress by pioglitazone in the heart of alloxan-induced diabetic rabbits. J Biomed Sci.

[216] Ahmed LA, Salem HA, Attia AS, Agha AM (2011). Pharmacological preconditioning with nicorandil and pioglitazone attenuates myocardial ischemia/reperfusion injury in rats. Eur J Pharm.

[217] Hu Q, Chen J, Jiang C, Liu H-F (2014). Effect of peroxisome proliferator-activated receptor gamma agonist on heart of rabbits with acute myocardial ischemia/reperfusion injury. Asian Pac J Trop Med.

[218] Singh RK, Gupta B, Tripathi K, Singh SK (2016). Anti oxidant potential of Metformin and Pioglitazone in Type 2 Diabetes Mellitus: Beyond their anti glycemic effect. Diabetes Metab Syndr.

[219] Taguchi A, Hayashi S (2011). Study of MDA-LDL by pioglitazone and pitavastatin in patients with type 2 diabetes. Nihon Rinsho.

[220] Erdmann E, Dormandy JA, Charbonnel B, Massi-Benedetti M, Moules IK, Skene AM (2007). The effect of pioglitazone on recurrent myocardial infarction in 2,445 patients with type 2 diabetes and previous myocardial infarction: results from the PROactive (PROactive 05) Study. J Am Coll Cardiol.

[221] de Jong M, van der Worp HB, van der Graaf Y, Visseren FLJ, Westerink J (2017). Pioglitazone and the secondary prevention of cardiovascular disease. A meta-analysis of randomized-controlled trials. Cardiovasc Diabetol.

[222] Zhu J, Yu X, Zheng Y, Li J, Wang Y, Lin Y (2020). Association of glucose-lowering medications with cardiovascular outcomes: an umbrella review and evidence map. Lancet Diabetes Endocrinol.

[223] Chen X, Yang L, Zhai S-D. Risk of cardiovascular disease and all-cause mortality among diabetic patients prescribed rosiglitazone or pioglitazone: a meta-analysis of retrospective cohort studies. Chin Med J. 2012;125:4301–6. 23217404

[224] Graham DJ, Ouellet-Hellstrom R, MaCurdy TE, Ali F, Sholley C, Worrall C (2010). Risk of acute myocardial infarction, stroke, heart failure, and death in elderly Medicare patients treated with rosiglitazone or pioglitazone. JAMA..

[225] Asensio-López MC, Lax A, Pascual-Figal DA, Valdés M, Sánchez-Más J (2011). Metformin protects against doxorubicin-induced cardiotoxicity: involvement of the adiponectin cardiac system. Free Radic Biol Med.

[226] Ashour AE, Sayed-Ahmed MM, Abd-Allah AR, Korashy HM, Maayah ZH, Alkhalidi H (2012). Metformin rescues the myocardium from doxorubicin-induced energy starvation and mitochondrial damage in rats. Oxid Med Cell Longev.

[227] Mohan M, Al-Talabany S, McKinnie A, Mordi IR, Singh JSS, Gandy SJ (2019). A randomized controlled trial of metformin on left ventricular hypertrophy in patients with coronary artery disease without diabetes: the MET-REMODEL trial. Eur Heart J.

[228] Greig D, Alcaino H, Castro PF, Garcia L, Verdejo HE, Navarro M (2011). Xanthine-oxidase inhibitors and statins in chronic heart failure: effects on vascular and functional parameters. J Heart Lung Transpl.

[229] Zhang Q, Qu H, Chen Y, Luo X, Chen C, Xiao B (2022). Atorvastatin induces mitochondria-dependent ferroptosis the modulation of Nrf2-xCT/GPx4 axis. Front Cell Dev Biol.

[230] Yu Y, Jin L, Zhuang Y, Hu Y, Cang J, Guo K (2018). Cardioprotective effect of rosuvastatin against isoproterenol-induced myocardial infarction injury in rats. Int J Mol Med.

[231] Zhou R, Xu Q, Zheng P, Yan L, Zheng J, Dai G (2008). Cardioprotective effect of fluvastatin on isoproterenol-induced myocardial infarction in rat. Eur J Pharm.

[232] Yu P, Zhang J, Ding Y, Chen D, Sun H, Yuan F (2022). Dexmedetomidine post-conditioning alleviates myocardial ischemia-reperfusion injury in rats by ferroptosis inhibition via SLC7A11/GPX4 axis activation. Hum Cell.

[233] Wang Z, Yao M, Jiang L, Wang L, Yang Y, Wang Q (2022). Dexmedetomidine attenuates myocardial ischemia/reperfusion-induced ferroptosis via AMPK/GSK-3β/Nrf2 axis. Biomed Pharmacother.

[234] Gao S, Ma G, Zhou L, Guan S, Zhang J (2022). Effects of dexmedetomidine pretreatment, posttreatment, and whole-course pumping on myocardial damage during cardiac valve replacement. Int Heart J.

[235] Chen W, Wang Y, Pan Z, Chen X, Luo D, Wang H (2021). Protective effects of dexmedetomidine on the ischemic myocardium in patients undergoing rheumatic heart valve replacement surgery. Exp Ther Med.

[236] Ji F, Li Z, Nguyen H, Young N, Shi P, Fleming N (2013). Perioperative dexmedetomidine improves outcomes of cardiac surgery. Circulation..

[237] Pitt B, Poole-Wilson PA, Segal R, Martinez FA, Dickstein K, Camm AJ (2000). Effect of losartan compared with captopril on mortality in patients with symptomatic heart failure: randomised trial-the Losartan Heart Failure Survival Study ELITE II. Lancet..

[238] Solomon SD, Skali H, Anavekar NS, Bourgoun M, Barvik S, Ghali JK (2005). Changes in ventricular size and function in patients treated with valsartan, captopril, or both after myocardial infarction. Circulation..

[239] Abdel-Wahab BA, Metwally ME, El-khawanki MM, Hashim AM (2014). Protective effect of captopril against clozapine-induced myocarditis in rats: role of oxidative stress, proinflammatory cytokines and DNA damage. Chem Biol Interact.

[240] Ibrahim MA, Ashour OM, Ibrahim YF, El-Bitar HI, Gomaa W, Abdel-Rahim SR (2009). Angiotensin-converting enzyme inhibition and angiotensin AT(1)-receptor antagonism equally improve doxorubicin-induced cardiotoxicity and nephrotoxicity. Pharm Res.

[241] Elfowiris A, Banigesh A (2022). Evaluation of antioxidant therapeutic value of ACE inhibitor as adjunct therapy on type 2 diabetes mellitus patients with cardiovascular disease. ACS Pharm Transl Sci.

[242] Asiri YA (2010). Probucol attenuates cyclophosphamide-induced oxidative apoptosis, p53 and Bax signal expression in rat cardiac tissues. Oxid Med Cell Longev.

[243] El-Demerdash E, Awad AS, Taha RM, El-Hady AM, Sayed-Ahmed MM (2005). Probucol attenuates oxidative stress and energy decline in isoproterenol-induced heart failure in rat. Pharm Res.

[244] Fu N, Yang S, Zhang J, Zhang P, Liang M, Cong H (2018). The efficacy of probucol combined with hydration in preventing contrast-induced nephropathy in patients with coronary heart disease undergoing percutaneous coronary intervention: a multicenter, prospective, randomized controlled study. Int Urol Nephrol.

[245] Yamashita S, Arai H, Bujo H, Masuda D, Ohama T, Ishibashi T (2021). Probucol trial for secondary prevention of atherosclerotic events in patients with coronary heart disease (PROSPECTIVE). J Atheroscler Thromb.

[246] He C, Zheng S, Luo Y, Wang B (2018). Exosome theranostics: biology and translational medicine. Theranostics.

[247] Song Y, Wang B, Zhu X, Hu J, Sun J, Xuan J (2021). Human umbilical cord blood-derived MSCs exosome attenuate myocardial injury by inhibiting ferroptosis in acute myocardial infarction mice. Cell Biol Toxicol.

[248] Zhang J-K, Zhang Z, Guo Z-A, Fu Y, Chen X-J, Chen W-J (2022). The BMSC-derived exosomal lncRNA Mir9-3hg suppresses cardiomyocyte ferroptosis in ischemia-reperfusion mice via the Pum2/PRDX6 axis. Nutr Metab Cardiovasc Dis.

[249] Zhao X, Si L, Bian J, Pan C, Guo W, Qin P (2022). Adipose tissue macrophage-derived exosomes induce ferroptosis via glutathione synthesis inhibition by targeting SLC7A11 in obesity-induced cardiac injury. Free Radic Biol Med.

[250] Liu C, Li B, Yan Q, Niu S, Zhao Y, Xiong C (2021). Protective effects and mechanisms of recombinant human glutathione peroxidase 4 on isoproterenol-induced myocardial ischemia injury. Oxid Med Cell Longev.

[251] Guo Z, Zhao M, Jia G, Ma R, Li M (2021). LncRNA PART1 alleviated myocardial ischemia/reperfusion injury via suppressing miR-503-5p/BIRC5 mediated mitochondrial apoptosis. Int J Cardiol.

[252] Ma S, Sun L, Wu W, Wu J, Sun Z, Ren J (2020). USP22 protects against myocardial ischemia-reperfusion injury via the SIRT1-p53/SLC7A11-dependent inhibition of ferroptosis-induced cardiomyocyte death. Front Physiol.

[253] Zhang C, Zeng L, Cai G, Zhu Y, Xiong Y, Zhan H (2022). miR-340-5p alleviates oxidative stress injury by targeting MyD88 in sepsis-induced cardiomyopathy. Oxid Med Cell Longev.

[254] Zhang W, Yang S, He H, Liu C, Chen W, Tang X (2009). Technology for improving the bioavailability of small molecules extracted from traditional Chinese medicines. Expert Opin Drug Deliv.

[255] Ishikawa K, Weber T, Hajjar RJ (2018). Human cardiac gene therapy. Circ Res.

[256] Greenberg B, Butler J, Felker GM, Ponikowski P, Voors AA, Desai AS (2016). Calcium upregulation by percutaneous administration of gene therapy in patients with cardiac disease (CUPID 2): a randomised, multinational, double-blind, placebo-controlled, phase 2b trial. Lancet..

