# Supplementary material for: Glutathione system enhancement for cardiac protection: pharmacological options against oxidative stress and ferroptosis
Source: Cell Death Dis. 2023 Feb 16;14(2):131. doi: 10.1038/s41419-023-05645-y (PMC9932120; doi:10.1038/s41419-023-05645-y)
Supplement: Supplementary file 1 — Supplementary Material [file 41419_2023_5645_MOESM1_ESM.docx]

**Supplementary Materials**

**Glutathione system enhancement for cardiac protection: pharmacological options against oxidative stress and ferroptosis**

*Mingyue Tan^1,†^, Yunfei Yin^1,†^, Xiao Ma^1,†^ , Jun Zhang^1^, Wanqian Pan^1^, Minghao Tan^2^,*

*Yongjian Zhao^1^, Tianke Yang^3,4^*, Tingbo Jiang^1,^*, Hongxia Li^1,^**

**Name and address of the institution**:

^1^ Department of Cardiology, The First Affiliated Hospital of Soochow University, 188 Shizi Street, Suzhou, Jiangsu 215006, P. R. China.

^2^ Department of Nursing, Tianfu College, Southwestern University of Finance and Economics, Chengdu, China

^3^ Department of Ophthalmology, Eye Institute, Eye & ENT Hospital, Fudan University, Shanghai, China.

^4^ Department of Ophthalmology, The First Affiliated Hospital of USTC, University of Science and Technology of China, Hefei, Anhui, 230001, China.

**Corresponding authors:**

**Hongxia Li**

Department of Cardiology, The First Affiliated Hospital of Soochow University, 188 Shizi Street, Suzhou, Jiangsu 215006, P. R. China. E-mail: **shrimp@suda.edu.cn**

**Tingbo Jiang**

Department of Cardiology, The First Affiliated Hospital of Soochow University, 188 Shizi Street, Suzhou, Jiangsu 215006, P. R. China E-mail: **18906201122@189.cn**

**Tianke Yang**

Department of Ophthalmology, Eye Institute, Eye & ENT Hospital, Fudan University, Shanghai, 200031, China.

Department of Ophthalmology, The First Affiliated Hospital of USTC, University of Science and Technology of China, Hefei, Anhui, 230001, China. E-mail: **gyytk94@sina.com**

^†^These authors contributed equally to this work.

**Running Title:** Glutathione system enhancement in cardiac injury therapy

**Table S1 Effects of raw material provision on the GSH system in the myocardium in a preclinical study**

| **Agent** | **Molecule** | **Modification in GSH system** | **Upstream targets** | **Ref** |
| --- | --- | --- | --- | --- |
| NAC | 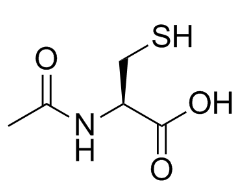 | GSH, GPX | n.a. | [1, 2] |
| Whey protein | - | GSH, GPX | n.a. | [3] |
| Glycine | 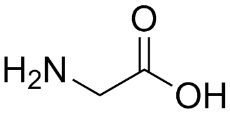 | GSH | n.a. | [4] |
| GlyNAC | - | GSH, GS, GCLC, GCLM | n.a. | [5, 6] |
| Glutamine | 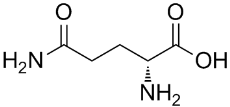 | GSH, GPX | n.a. | [7, 8] |
| Selenium | - | GSH, GPX | n.a. | [9] |

**Table S2 Effects of natural** **medicine monomers on the GSH system in the myocardium in a preclinical study**

| **Agent** | **Molecule** | **Modification in GSH system** | **Upstream targets** | **Ref** |
| --- | --- | --- | --- | --- |
| **Flavonoids** |  |  |  |  |
| Quercetin | 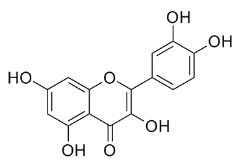 | GSH, GPX, GCL, GR | P13K/Akt, PPAR-γ, Nrf2 | [10-12] |
| Catechin | 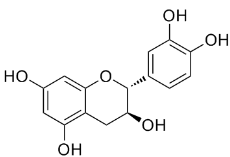 | GSH, GST | n.a. | [13] |
| EC | 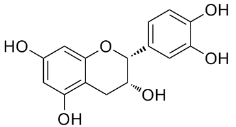 | GSH, GPX, GR | n.a. | [14] |
| EGCG | 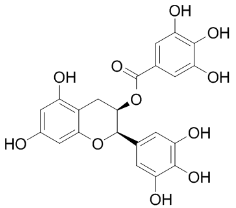 | GSH, GPX, GST, GR | n.a. | [15] |
| Genistein | 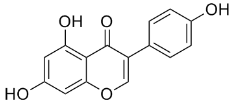 | GSH, GPX | Nrf2 | [16, 17] |
| **Terpenoids** | | | | |
| β‐carotene | 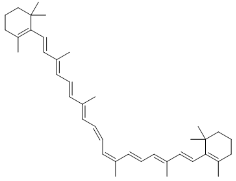 | GSH, GPX, GR | n.a. | [18] |
| Lycopene | 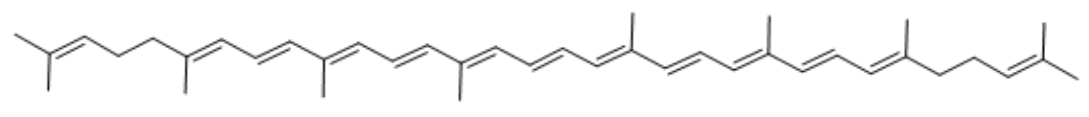 | GSH, GPX | Akt/Nrf2 | [19, 20] |
| Tan IIA | 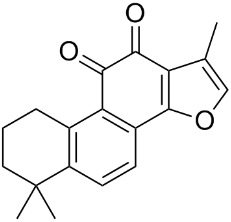 | GSH, GPX, GCL | Nrf2 | [21, 22] |
| **Phenolic acid** | | | | |
| Ferulic acid | 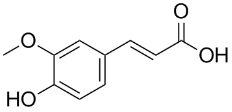 | GSH, GPX | AMPKα2, Nrf2 | [23, 24] |
| Caffeic acid | 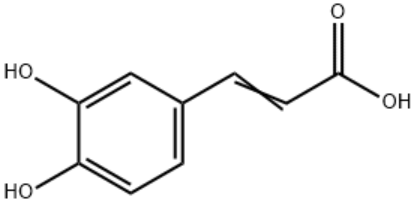 | GSH, GPX, GST, GR | n.a. | [25] |
| Chlorogenic acid | 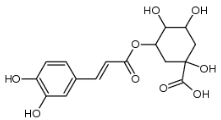 | GSH, GPX, GST | n.a. | [26] |
| Cinnamic acid | 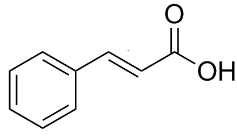 | GSH | n.a. | [27] |
| Ellagic acid | 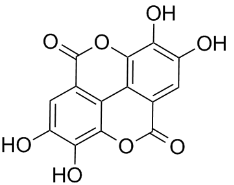 | GSH, GPX | n.a. | [28, 29] |
| Urolithin A | 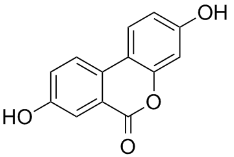 | GSH | n.a. | [30] |
| Syringic acid | 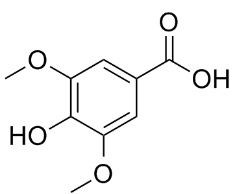 | GSH, GPX, GST | n.a. | [31] |
| Danshensu | 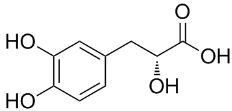 | GSH, GPX, GST, GR | Nrf2 | [32] |
| **Quinones** |  |  |  |  |
| CoQ10 | 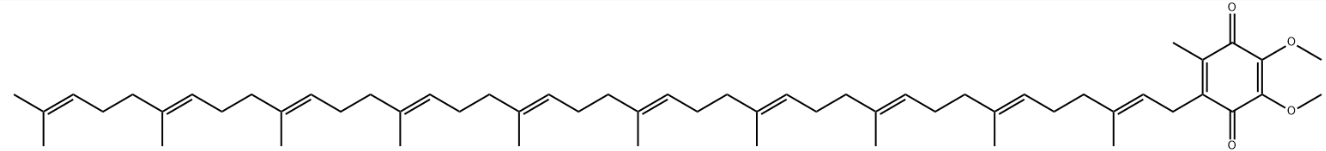 | GSH | Nrf2 | [33, 34] |
| Thy | 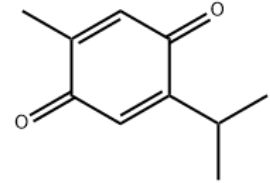 | GSH, GPX, GST | n.a. | [35] |
| β-LAPachone | 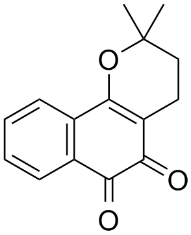 | GPX, GST | Nrf2 | [36] |
| Aloin | 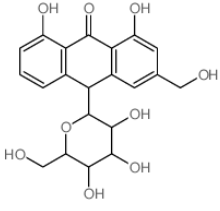 | GSH | n.a. | [37] |
| **Others** | | | | |
| Curcumin | 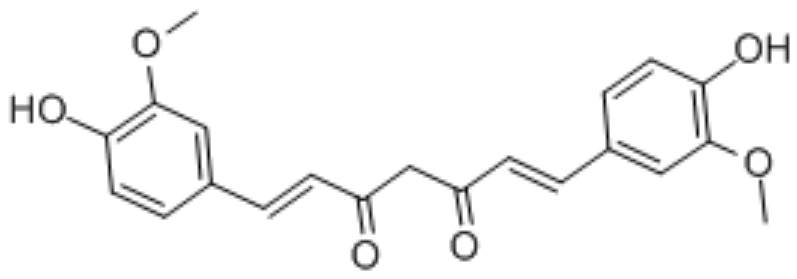 | GSH, GPX, GR | Nrf2, Sirt3, PPAR-γ | [38-40] |
| RSV | 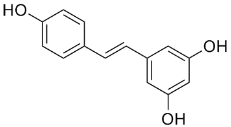 | GSH, SLC7A11, GPX, GR | Nrf2, AMPK, Sirt1, miR-149, KAT5 | [41-46] |

**Table S3 Effects of synthetic medicines on the GSH system in the myocardium in a preclinical study**

| **Agent** | **Molecule** | **Mode of action** | **Modification in GSH system** | **Ref** |
| --- | --- | --- | --- | --- |
| **Activating upstream targets of the GSH system** | | | | |
| Melatonin | 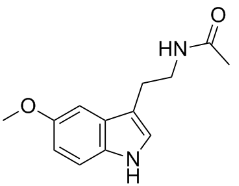 | Nrf2 | GSH, GPX | [47, 48] |
| Trimetazidine | 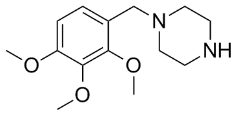 |  | GSH, GPX | [49-52]. |
| Pioglitazone | 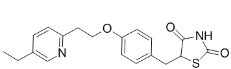 | PPAR-γ | GSH, GPX, GR | [53] |
| Rosiglitazone | 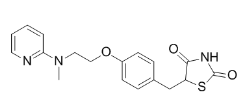 |  | GSH, GPX | [54] |
| Metformin | 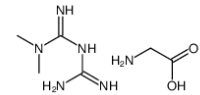 | AMPK | GSH, GPX | [55, 56] |
| **Enhancing GSH synthesis or / and metabolism** | | | | |
| Atorvastatin | 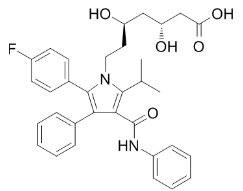 | Upregulating SLC7A11 | GSH, GPX4 | [57] |
| Dexmedetomidine | 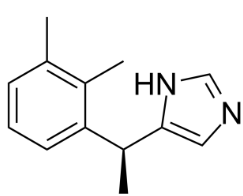 |  | GSH, GPX4 | [58] |
| **Increasing GSH or / and GSH-dependent antioxidant enzymes directly.** | | | | |
| Captopril | 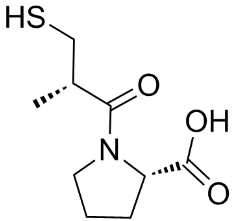 |  | GSH, GPX | [59, 60] |
| Probucol | 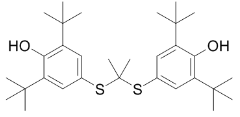 |  | GSH, GPX | [61-64] |

NAC: N-acetyl-cysteine; GSH: glutathione disulfide; GSSG: oxidized glutathione; GPX: glutathione peroxidase; GS: glutathione synthetase; GCLC: glutamate-cysteine ligase catalytic subunit; GCLM: glutamate-cysteine ligase modifier subunit; EC: epicatechin; EGCG: epigallocatechin-3-gallate; PPAR-γ: peroxisome proliferator-activated receptor-gamma; AKT: phosphoinositide-3-kinase; Nrf2: erythrocyte nuclear factor 2; GR: glutathione reductase; Tan IIA: tanshinone IIA; CoQ10: coenzyme Q10; Thy: thymoquinone; AMPK: Adenosine 5‘-monophosphate (AMP)-activated protein kinase; RSV: resveratrol.

**References**

1. Rushworth GF, Megson IL. Existing and potential therapeutic uses for N-acetylcysteine: the need for conversion to intracellular glutathione for antioxidant benefits. Pharmacol Ther. 2014;141:150-9.

2. Basha RH, Priscilla DH. An in vivo and in vitro study on the protective effects of N-acetylcysteine on mitochondrial dysfunction in isoproterenol treated myocardial infarcted rats. Exp Toxicol Pathol. 2013;65:7-14.

3. Bartfay WJ, Davis MT, Medves JM, Lugowski S. Milk whey protein decreases oxygen free radical production in a murine model of chronic iron-overload cardiomyopathy. Can J Cardiol. 2003;19:1163-8.

4. Zhang Y, Lv SJ, Yan H, Wang L, Liang GP, Wan QX, et al. Effects of glycine supplementation on myocardial damage and cardiac function after severe burn. Burns. 2013;39:729-35.

5. Cieslik KA, Sekhar RV, Granillo A, Reddy A, Medrano G, Heredia CP, et al. Improved Cardiovascular Function in Old Mice After N-Acetyl Cysteine and Glycine Supplemented Diet: Inflammation and Mitochondrial Factors. J Gerontol A Biol Sci Med Sci. 2018;73:1167-77.

6. Kumar P, Osahon OW, Sekhar RV. GlyNAC (Glycine and N-Acetylcysteine) Supplementation in Mice Increases Length of Life by Correcting Glutathione Deficiency, Oxidative Stress, Mitochondrial Dysfunction, Abnormalities in Mitophagy and Nutrient Sensing, and Genomic Damage. Nutrients. 2022;14.

7. S HSK, Anandan R. Biochemical studies on the cardioprotective effect of glutamine on tissue antioxidant defense system in isoprenaline-induced myocardial infarction in rats. J Clin Biochem Nutr. 2007;40:49-55.

8. Todorova V, Vanderpool D, Blossom S, Nwokedi E, Hennings L, Mrak R, et al. Oral glutamine protects against cyclophosphamide-induced cardiotoxicity in experimental rats through increase of cardiac glutathione. Nutrition (Burbank, Los Angeles County, Calif). 2009;25:812-7.

9. Tanguy S, Morel S, Berthonneche C, Toufektsian M-C, de Lorgeril M, Ducros V, et al. Preischemic selenium status as a major determinant of myocardial infarct size in vivo in rats. Antioxidants & Redox Signaling. 2004;6:792-6.

10. Liu X, Yu Z, Huang X, Gao Y, Wang X, Gu J, et al. Peroxisome proliferator-activated receptor γ (PPARγ) mediates the protective effect of quercetin against myocardial ischemia-reperfusion injury via suppressing the NF-κB pathway. American Journal of Translational Research. 2016;8:5169-86.

11. El-Sayed SS, Shahin RM, Fahmy A, Elshazly SM. Quercetin ameliorated remote myocardial injury induced by renal ischemia/reperfusion in rats: Role of Rho-kinase and hydrogen sulfide. Life Sci. 2021;287:120144.

12. Liu H, Guo X, Chu Y, Lu S. Heart protective effects and mechanism of quercetin preconditioning on anti-myocardial ischemia reperfusion (IR) injuries in rats. Gene. 2014;545:149-55.

13. Saleh Ahmed AS. Potential protective effect of catechin on doxorubicin-induced cardiotoxicity in adult male albino rats. Toxicol Mech Methods. 2022;32.

14. Prince PSM. A biochemical, electrocardiographic, electrophoretic, histopathological and in vitro study on the protective effects of (-)epicatechin in isoproterenol-induced myocardial infarcted rats. Eur J Pharmacol. 2011;671.

15. Devika PT, Stanely Mainzen Prince P. Protective effect of (-)-epigallocatechin-gallate (EGCG) on lipid peroxide metabolism in isoproterenol induced myocardial infarction in male Wistar rats: a histopathological study. Biomed Pharmacother. 2008;62:701-8.

16. Bai Z, Wang Z. Genistein protects against doxorubicin-induced cardiotoxicity through Nrf-2/HO-1 signaling in mice model. Environ Toxicol. 2019;34:645-51.

17. Jia Q, Wang Y, Liu X, Ma S, Yang R. [Effects of genistein on Nrf2/HO-1 pathway in myocardial tissues of diabetic rats]. Zhong Nan Da Xue Xue Bao Yi Xue Ban. 2019;44:850-6.

18. Maritim A, Dene BA, Sanders RA, Watkins JB. Effects of beta-carotene on oxidative stress in normal and diabetic rats. J Biochem Mol Toxicol. 2002;16:203-8.

19. Zheng S, Deng Z, Chen F, Zheng L, Pan Y, Xing Q, et al. Synergistic antioxidant effects of petunidin and lycopene in H9c2 cells submitted to hydrogen peroxide: Role of Akt/Nrf2 pathway. J Food Sci. 2020;85:1752-63.

20. Abdel-Daim MM, Eltaysh R, Hassan A, Mousa SA. Lycopene Attenuates Tulathromycin and Diclofenac Sodium-Induced Cardiotoxicity in Mice. Int J Mol Sci. 2018;19.

21. Guo Z, Yan M, Chen L, Fang P, Li Z, Wan Z, et al. Nrf2-dependent antioxidant response mediated the protective effect of tanshinone IIA on doxorubicin-induced cardiotoxicity. Exp Ther Med. 2018;16:3333-44.

22. Hu H, Zhai C, Qian G, Gu A, Liu J, Ying F, et al. Protective effects of tanshinone IIA on myocardial ischemia reperfusion injury by reducing oxidative stress, HMGB1 expression, and inflammatory reaction. Pharmaceutical Biology. 2015;53:1752-8.

23. Liu X, Qi K, Gong Y, Long X, Zhu S, Lu F, et al. Ferulic Acid Alleviates Myocardial Ischemia Reperfusion Injury Via Upregulating AMPKα2 Expression-Mediated Ferroptosis Depression. Journal of Cardiovascular Pharmacology. 2021;79:489-500.

24. Yeh C-T, Ching L-C, Yen G-C. Inducing gene expression of cardiac antioxidant enzymes by dietary phenolic acids in rats. J Nutr Biochem. 2009;20:163-71.

25. Kumaran KS, Prince PSM. Protective effect of caffeic acid on cardiac markers and lipid peroxide metabolism in cardiotoxic rats: an in vivo and in vitro study. Metabolism. 2010;59:1172-80.

26. Akila P, Vennila L. Chlorogenic acid a dietary polyphenol attenuates isoproterenol induced myocardial oxidative stress in rat myocardium: An in vivo study. Biomedicine & Pharmacotherapy = Biomedecine & Pharmacotherapie. 2016;84:208-14.

27. Anupama N, Preetha Rani MR, Shyni GL, Raghu KG. Glucotoxicity results in apoptosis in H9c2 cells via alteration in redox homeostasis linked mitochondrial dynamics and polyol pathway and possible reversal with cinnamic acid. Toxicol In Vitro. 2018;53:178-92.

28. Chao P-C, Hsu C-C, Yin M-C. Anti-inflammatory and anti-coagulatory activities of caffeic acid and ellagic acid in cardiac tissue of diabetic mice. Nutr Metab (Lond). 2009;6:33.

29. Yüce A, Ateşşahin A, Ceribaşi AO, Aksakal M. Ellagic acid prevents cisplatin-induced oxidative stress in liver and heart tissue of rats. Basic & Clinical Pharmacology & Toxicology. 2007;101:345-9.

30. Albasher G, Alkahtani S, Al-Harbi LN. Urolithin A prevents streptozotocin-induced diabetic cardiomyopathy in rats by activating SIRT1. Saudi J Biol Sci. 2022;29:1210-20.

31. Sammeturi M, Shaik AH, Bongu SBR, Cheemanapalli S, Mohammad A, Kodidhela LD. Protective effects of syringic acid, resveratrol and their combination against isoprenaline administered cardiotoxicity in wistar rats. Saudi J Biol Sci. 2019;26:1429-35.

32. Li H, Xie Y-H, Yang Q, Wang S-W, Zhang B-L, Wang J-B, et al. Cardioprotective effect of paeonol and danshensu combination on isoproterenol-induced myocardial injury in rats. PLoS One. 2012;7:e48872.

33. Ghule AE, Kulkarni CP, Bodhankar SL, Pandit VA. Effect of pretreatment with coenzyme Q10 on isoproterenol-induced cardiotoxicity and cardiac hypertrophy in rats. Curr Ther Res Clin Exp. 2009;70:460-71.

34. Li X, Zhan J, Hou Y, Hou Y, Chen S, Luo D, et al. Coenzyme Q10 Regulation of Apoptosis and Oxidative Stress in HO Induced BMSC Death by Modulating the Nrf-2/NQO-1 Signaling Pathway and Its Application in a Model of Spinal Cord Injury. Oxidative Medicine and Cellular Longevity. 2019;2019:6493081.

35. Danaei GH, Memar B, Ataee R, Karami M. Protective effect of thymoquinone, the main component of , against diazinon cardio-toxicity in rats. Drug Chem Toxicol. 2019;42:585-91.

36. Nazari Soltan Ahmad S, Sanajou D, Kalantary-Charvadeh A, Hosseini V, Roshangar L, Khojastehfard M, et al. beta-LAPachone ameliorates doxorubicin-induced cardiotoxicity via regulating autophagy and Nrf2 signalling pathways in mice. Basic Clin Pharmacol Toxicol. 2020;126:364-73.

37. Birari L, Wagh S, Patil KR, Mahajan UB, Unger B, Belemkar S, et al. Aloin alleviates doxorubicin-induced cardiotoxicity in rats by abrogating oxidative stress and pro-inflammatory cytokines. Cancer Chemother Pharmacol. 2020;86:419-26.

38. Mishra P, Paital B, Jena S, Swain SS, Kumar S, Yadav MK, et al. Possible activation of NRF2 by Vitamin E/Curcumin against altered thyroid hormone induced oxidative stress via NFĸB/AKT/mTOR/KEAP1 signalling in rat heart. Sci Rep. 2019;9:7408.

39. Wang R, Zhang JY, Zhang M, Zhai MG, Di SY, Han QH, et al. Curcumin attenuates IR-induced myocardial injury by activating SIRT3. Eur Rev Med Pharmacol Sci. 2018;22:1150-60.

40. Chen R, Peng X, Du W, Wu Y, Huang B, Xue L, et al. Curcumin attenuates cardiomyocyte hypertrophy induced by high glucose and insulin via the PPARγ/Akt/NO signaling pathway. Diabetes Res Clin Pract. 2015;108:235-42.

41. Ibrahim KA, Abdelgaid HA, Eleyan M, Mohamed RA, Gamil NM. Resveratrol alleviates cardiac apoptosis following exposure to fenitrothion by modulating the sirtuin1/c-Jun N-terminal kinases/p53 pathway through pro-oxidant and inflammatory response improvements: In vivo and in silico studies. Life Sciences. 2022;290:120265.

42. Cheng L, Jin Z, Zhao R, Ren K, Deng C, Yu S. Resveratrol attenuates inflammation and oxidative stress induced by myocardial ischemia-reperfusion injury: role of Nrf2/ARE pathway. Int J Clin Exp Med. 2015;8:10420-8.

43. Xu G, Zhao X, Fu J, Wang X. Resveratrol increase myocardial Nrf2 expression in type 2 diabetic rats and alleviate myocardial ischemia/reperfusion injury (MIRI). Ann Palliat Med. 2019;8:565-75.

44. Guo S, Yao Q, Ke Z, Chen H, Wu J, Liu C. Resveratrol attenuates high glucose-induced oxidative stress and cardiomyocyte apoptosis through AMPK. Mol Cell Endocrinol. 2015;412:85-94.

45. Wang X, Simayi A, Fu J, Zhao X, Xu G. Resveratrol mediates the miR-149/HMGB1 axis and regulates the ferroptosis pathway to protect myocardium in endotoxemia mice. Am J Physiol Endocrinol Metab. 2022.

46. Liu J, Zhang M, Qin C, Wang Z, Chen J, Wang R, et al. Resveratrol Attenuate Myocardial Injury by Inhibiting Ferroptosis Inducing KAT5/GPX4 in Myocardial Infarction. Frontiers In Pharmacology. 2022;13:906073.

47. Zhi W, Li K, Wang H, Lei M, Guo Y. Melatonin elicits protective effects on OGD/R‑insulted H9c2 cells by activating PGC‑1α/Nrf2 signaling. Int J Mol Med. 2020;45:1294-304.

48. Cai J, Yang J, Chen X, Zhang H, Zhu Y, Liu Q, et al. Melatonin ameliorates trimethyltin chloride-induced cardiotoxicity: The role of nuclear xenobiotic metabolism and Keap1-Nrf2/ARE axis-mediated pyroptosis. Biofactors. 2022;48:481-97.

49. Zhang H, Liu M, Zhang Y, Li X. Trimetazidine Attenuates Exhaustive Exercise-Induced Myocardial Injury in Rats via Regulation of the Nrf2/NF-κB Signaling Pathway. Frontiers In Pharmacology. 2019;10:175.

50. Wu S, Chang G, Gao L, Jiang D, Wang L, Li G, et al. Trimetazidine protects against myocardial ischemia/reperfusion injury by inhibiting excessive autophagy. J Mol Med (Berl). 2018;96:791-806.

51. Eid BG, El-Shitany NAE-A, Neamatallah T. Trimetazidine improved adriamycin-induced cardiomyopathy by downregulating TNF-α, BAX, and VEGF immunoexpression via an antioxidant mechanism. Environ Toxicol. 2021;36:1217-25.

52. Ramezani-Aliakbari F, Badavi M, Dianat M, Mard SA, Ahangarpour A. The Effects of Trimetazidine on QT-interval Prolongation and Cardiac Hypertrophy in Diabetic Rats. Arq Bras Cardiol. 2019;112:173-8.

53. Gumieniczek A. Modification of oxidative stress by pioglitazone in the heart of alloxan-induced diabetic rabbits. J Biomed Sci. 2005;12:531-7.

54. Zhang L, Wu P, Zhang L, SreeHarsha N, Mishra A, Su X. Ameliorative effect of rosiglitazone, a peroxisome proliferator gamma agonist on adriamycin-induced cardio toxicity via suppressing oxidative stress and apoptosis. IUBMB Life. 2020;72:607-15.

55. Asensio-López MC, Lax A, Pascual-Figal DA, Valdés M, Sánchez-Más J. Metformin protects against doxorubicin-induced cardiotoxicity: involvement of the adiponectin cardiac system. Free Radical Biology & Medicine. 2011;51:1861-71.

56. Ashour AE, Sayed-Ahmed MM, Abd-Allah AR, Korashy HM, Maayah ZH, Alkhalidi H, et al. Metformin rescues the myocardium from doxorubicin-induced energy starvation and mitochondrial damage in rats. Oxidative Medicine and Cellular Longevity. 2012;2012:434195.

57. Ning D, Yang X, Wang T, Jiang Q, Yu J, Wang D. Atorvastatin treatment ameliorates cardiac function and remodeling induced by isoproterenol attack through mitigation of ferroptosis. Biochem Biophys Res Commun. 2021;574:39-47.

58. Yu P, Zhang J, Ding Y, Chen D, Sun H, Yuan F, et al. Dexmedetomidine post-conditioning alleviates myocardial ischemia-reperfusion injury in rats by ferroptosis inhibition via SLC7A11/GPX4 axis activation. Hum Cell. 2022;35:836-48.

59. Abdel-Wahab BA, Metwally ME, El-khawanki MM, Hashim AM. Protective effect of captopril against clozapine-induced myocarditis in rats: role of oxidative stress, proinflammatory cytokines and DNA damage. Chem Biol Interact. 2014;216:43-52.

60. Ibrahim MA, Ashour OM, Ibrahim YF, El-Bitar HI, Gomaa W, Abdel-Rahim SR. Angiotensin-converting enzyme inhibition and angiotensin AT(1)-receptor antagonism equally improve doxorubicin-induced cardiotoxicity and nephrotoxicity. Pharmacol Res. 2009;60:373-81.

61. Asiri YA. Probucol attenuates cyclophosphamide-induced oxidative apoptosis, p53 and Bax signal expression in rat cardiac tissues. Oxidative Medicine and Cellular Longevity. 2010;3:308-16.

62. Li T, Danelisen I, Belló-Klein A, Singal PK. Effects of probucol on changes of antioxidant enzymes in adriamycin-induced cardiomyopathy in rats. Cardiovasc Res. 2000;46:523-30.

63. El-Demerdash E, Awad AS, Taha RM, El-Hady AM, Sayed-Ahmed MM. Probucol attenuates oxidative stress and energy decline in isoproterenol-induced heart failure in rat. Pharmacol Res. 2005;51:311-8.

64. Kaul N, Siveski-Iliskovic N, Thomas TP, Hill M, Khaper N, Singal PK. Probucol improves antioxidant activity and modulates development of diabetic cardiomyopathy. Nutrition (Burbank, Los Angeles County, Calif). 1995;11:551-4.
